# Quantitative spatial analysis of chromatin biomolecular condensates using cryoelectron tomography

**DOI:** 10.1073/pnas.2426449122

**Published:** 2025-05-06

**Authors:** Huabin Zhou, Joshua Hutchings, Momoko Shiozaki, Xiaowei Zhao, Lynda K. Doolittle, Shixin Yang, Rui Yan, Nikki Jean, Margot Riggi, Zhiheng Yu, Elizabeth Villa, Michael K. Rosen

**Affiliations:** ^a^Department of Biophysics, University of Texas Southwestern Medical Center, Dallas, TX 75390; ^b^HHMI, University of Texas Southwestern Medical Center, Dallas, TX 75390; ^c^School of Biological Sciences, University of California, San Diego, La Jolla, CA 92093; ^d^Janelia Research Campus, HHMI, Ashburn, VA 20147; ^e^Research Department Cell and Virus Structure, Max Planck Institute for Biochemistry, Martinsried/Munich D-82152, Germany; ^f^HHMI, University of California, San Diego, La Jolla, CA 92093

**Keywords:** cryoelectron tomography, biomolecular condensate, chromatin, phase separation, nucleosome

## Abstract

Phase separation is an important mechanism to generate certain biomolecular condensates and organize the cell interior. Condensate formation and function remain incompletely understood due to difficulties in visualizing the condensate interior at high resolution. Here, we analyzed the structure of biochemically reconstituted chromatin condensates through cryoelectron tomography. We found that traditional blotting methods of sample preparation were inadequate, and high-pressure freezing plus focused ion beam milling was essential to maintain condensate integrity. To identify densely packed molecules within the condensate, we integrated deep learning–based segmentation with context-aware template matching. Our approaches were developed on chromatin condensates and were also effective on condensed regions of in situ native chromatin. Using these methods, we determined the average structure of nucleosomes to 6.1 and 12 Å resolution in reconstituted and native systems, respectively, found that nucleosomes form heterogeneous interaction networks in both cases, and gained insight into the molecular origins of surface tension in chromatin condensates. Our methods should be applicable to biomolecular condensates containing large and distinctive components in both biochemical reconstitutions and certain cellular systems.

## Significance

Biomolecular condensates play important roles in cellular organization and function, but their internal structures remain poorly understood due to limitations in imaging techniques. This is true for both cellular condensates and those reconstituted biochemically. We developed a workflow that preserves the integrity of biochemically reconstituted condensates and enables high-resolution structural analysis. Applied to chromatin condensates and native chromatin, we determined structures of both the basic nucleosome units and their higher-order interaction networks, revealing similar network heterogeneity despite the vastly different chromatin fiber lengths in the two systems. Our workflow should be extendable to other condensates composed of large, recognizable components and paves the way for understanding the structural basis of condensate behaviors in normal biology and disease.

Biomolecular condensates are increasingly recognized for their important roles in myriad biological processes, ranging from gene expression and signal transduction to stress responses (1–3). Dysregulation of condensates is implicated in diseases including neurodegenerative disorders (e.g., amyotrophic lateral sclerosis), cancer, and viral infection (4–6). The internal structure and dynamic rearrangements of condensates are believed to play pivotal roles in the normal functions of condensates and in disease progression (1, 2).

Analogous to traditional structural analyses of molecular machines, direct visualization of components within condensates could provide substantial insights into the mechanisms by which condensates form, respond to signals, and function. This goal encompasses characterizing the conformations of individual molecules and their discrete complexes and also understanding the higher-order organization of these components, encompassing their positions, orientations, and interactions. Notably, this latter goal is different from most NMR, crystallographic, and single-particle cryoelectron microscopy (cryo-EM) analyses, which generally seek to understand the average structure of individual components at high resolution rather than the spatial arrangements of molecules in the collection.

Biochemical reconstitution of condensates offers a controlled environment that simplifies these complex systems, allowing for detailed studies of their activities and the derivation of underlying principles applicable to more complex in vivo systems (7–10). Structural studies of such reconstituted systems are particularly promising as they provide a precise understanding of structure–function relationships in a well-defined context where all component parts are known a priori.

Cryoelectron tomography (cryo-ET) is a potentially powerful technique for structural analyses of condensates because it enables visualization of all individual particles within a field of view (11). Particles can be computationally combined to yield an average structure, and also their spatial arrangements can be analyzed to yield information on higher-order organization. However, unique and generic challenges arise when studying condensates with cryo-ET. Many condensates behave as dynamic liquids, in which molecules interact weakly and transiently (1–3). This property makes them particularly susceptible to perturbations during sample preparation, especially in biochemically reconstituted systems (12, 13). The high density of molecules within condensates can also obscure individual molecular details and produce overlapping signals in low electron dosed, noisy tomograms. Furthermore, to achieve high-resolution reconstructions of individual molecules/complexes, traditional single-particle cryo-EM approaches typically discard the vast majority of particles (14), and cryo-ET approaches compensate for missing wedge artifacts by averaging particles in different orientations (11, 15). However, these procedures are inappropriate when a key goal of a study is to identify all particles in a sample and understand their spatial organization. Because of these issues, while a number of studies of both biochemically reconstituted (16–19) and cellular (20–22) condensates have substantially benefitted from cryo-ET, these analyses have not yet yielded high-resolution structures of condensate components or a quantitative understanding of the network of interactions between molecules.

We sought to address these challenges through cryo-ET studies of condensates formed by polynucleosome arrays, which model cellular chromatin from the eukaryotic nucleus (13, 23, 24). We selected this condensate system for several reasons. First, chromatin condensates are representative of the many condensates that form through multivalency-driven liquid–liquid phase separation (LLPS) (23, 25). Second, they present significant technical advantages for cryo-ET, as individual nucleosomes are flattened discs of known structure, consisting of an ~120 kDa proteinaceous core wrapped twice by ~96 kDa (~147 base pairs) of double-stranded DNA (26). Thus, nucleosomes are easily distinguished and identified within tomograms due to their large, unique electron-dense shape. Finally, both the structure of individual nucleosomes and their higher-order organization are important for numerous genomic functions (27–29), and chromatin condensates represent a powerful biomimetic system to understand how nuclear biochemistry is affected by the chromatin environment (23). Several studies have investigated the structure of native chromatin fibers using cryo-EM. However, most of these have concentrated on the conformations of individual nucleosomes or fibers rather than the higher-order organization of these units or the network of interactions between them (17, 30–34).

Using chromatin condensates, we developed a pipeline spanning from sample preparation to image analysis that enables structural investigations of the condensate interior. The pipeline is effective for both biochemically reconstituted chromatin condensates and native chromatin in isolated mammalian cell nuclei and intact mammalian cells. Using it, we determined the average structure of nucleosomes to 6.1 Å and 12 Å resolution from the tomography data in reconstituted and native systems, respectively, and show that nucleosomes have a nearly random orientation distribution in both cases. Our methodology should be applicable to reconstituted condensates and to certain cellular condensates, in both cases where components are large and distinctive.

## Results

### Blotting and Self-Wicking Distort Condensate Morphologies.

**Fig. 1.**
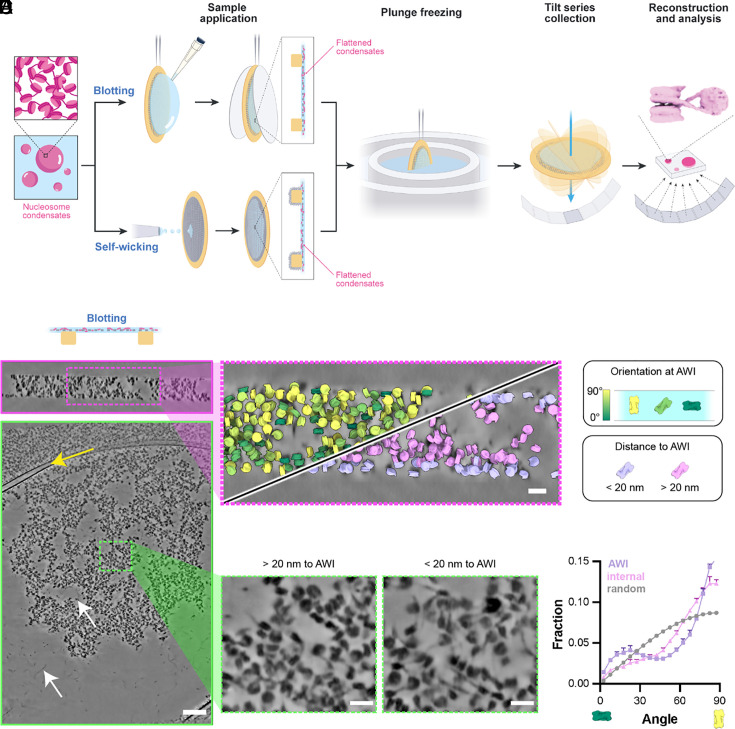
Morphological distortions in condensates by blotting and self-wicking techniques. (*A*) Diagram depicting blotting- and self-wicking-based sample preparation for cryo-ET. (*B*) Orthogonal cross-sections of chromatin condensates processed using the blotting technique. The yellow arrow indicates carbon support film on the grids. White arrows indicate exposed nucleosomes and bare DNA regions. (Scale bar, 100 nm.) X-Y and X-Z views are shown with green and magenta outlines, respectively. (*C*) Zoom-in the X-Z view of the tomogram, with the assigned nucleosomes colored by the relative orientation [angle between the plane of air–water interface (AWI) and the plane of the nucleosome] (*Top Left*) or distance (*Bottom Right*) to the AWI. (Scale bar, 20 nm.) (*D*) Representative sections demonstrating chromatin condensates located close to and away from the AWI. (Scale bars, 20 nm.) (*E*) Distribution of angle between the normal to the nucleosome plane and plane of AWI at the AWI and in the core of the condensate.

In vitro reconstituted nucleosome arrays (*SI Appendix*, Fig. S1*A*) undergo phase separation under physiologic salt conditions, driven primarily by screening of the negative charge of the DNA backbone and mediated by histone–DNA and histone–histone interactions (35–37). To visualize condensates using cryo-ET, the sample must be sufficiently thin to allow electron transmission. Initially, we applied standard blotting techniques, where solutions containing chromatin condensates were pipetted onto grids, and excess liquid was removed with filter paper to create a thin layer (Fig. 1*A*). Subsequent plunge freezing and tilt-series collection enabled reconstruction of tomograms with high contrast (Fig. 1*B*). However, this process substantially distorted the chromatin condensate structure. The droplets were compressed into a thin layer, which, coupled with capillary forces, caused significant deformation (Fig. 1*B*). The droplets were no longer round, contained numerous internal cavities, and consistently adhered to the carbon support on the grid. Extensive naked DNA was also observed, indicating that nucleosomes had been disassembled. These artifacts compromised the reliability of the structural data. Attempts to mitigate damage using a one-sided blotting technique were unsuccessful; droplets still adhered to the carbon in unusual shapes (*SI Appendix F*ig. S1 *B* and *C*).

Subsequently, we explored the self-wicking method (38), in which a small volume of sample is sprayed on the grid and nanofibers on the grid then extract excess buffer to reduce specimen thickness (Fig. 1*A*). This approach presented similar artifacts; droplets continued to adhere to the carbon support, were compressed into thin layers, and exposed significant amounts of naked DNA (*SI Appendix*, Fig. S1 *D* and *E*). As described below, blotting and self-wicking also altered the density of nucleosomes in the condensates.

An additional problem with both the blotting and self-wicking approaches is that nucleosomes at the AWI displayed a marked orientational bias. Context aware template matching (CATM; detailed below) analysis of nucleosome orientations revealed that nucleosomes throughout the sample had an orientation distribution that is highly nonrandom, contrary to expectations for an isotropic condensate (see below). Those at the AWI present disproportionately have their faces parallel to the interface (Fig. 1 *C*–*E* and *SI Appendix*, Fig. S1 *E* and *F*).

To further investigate this bias, we blotted non-phase-separated samples composed of mononucleosomes and collected tomograms (*SI Appendix*, Fig. S2*A*). The resulting images confirmed that nucleosomes were also predominantly trapped at the AWI in a face-on orientation with unwrapped nucleosomes (*SI Appendix*, Fig. S2 *B* and *C*). Preferred orientation due to the AWI is a well-documented issue in single-particle cryo-EM (39); our results further highlight the challenges posed by these sample preparation methods in preserving native higher-order organization of biochemically reconstituted condensates.

### Preservation of Droplet Morphology Via High-Pressure Freezing and Cryofocused Ion Beam Milling.

**Fig. 2.**
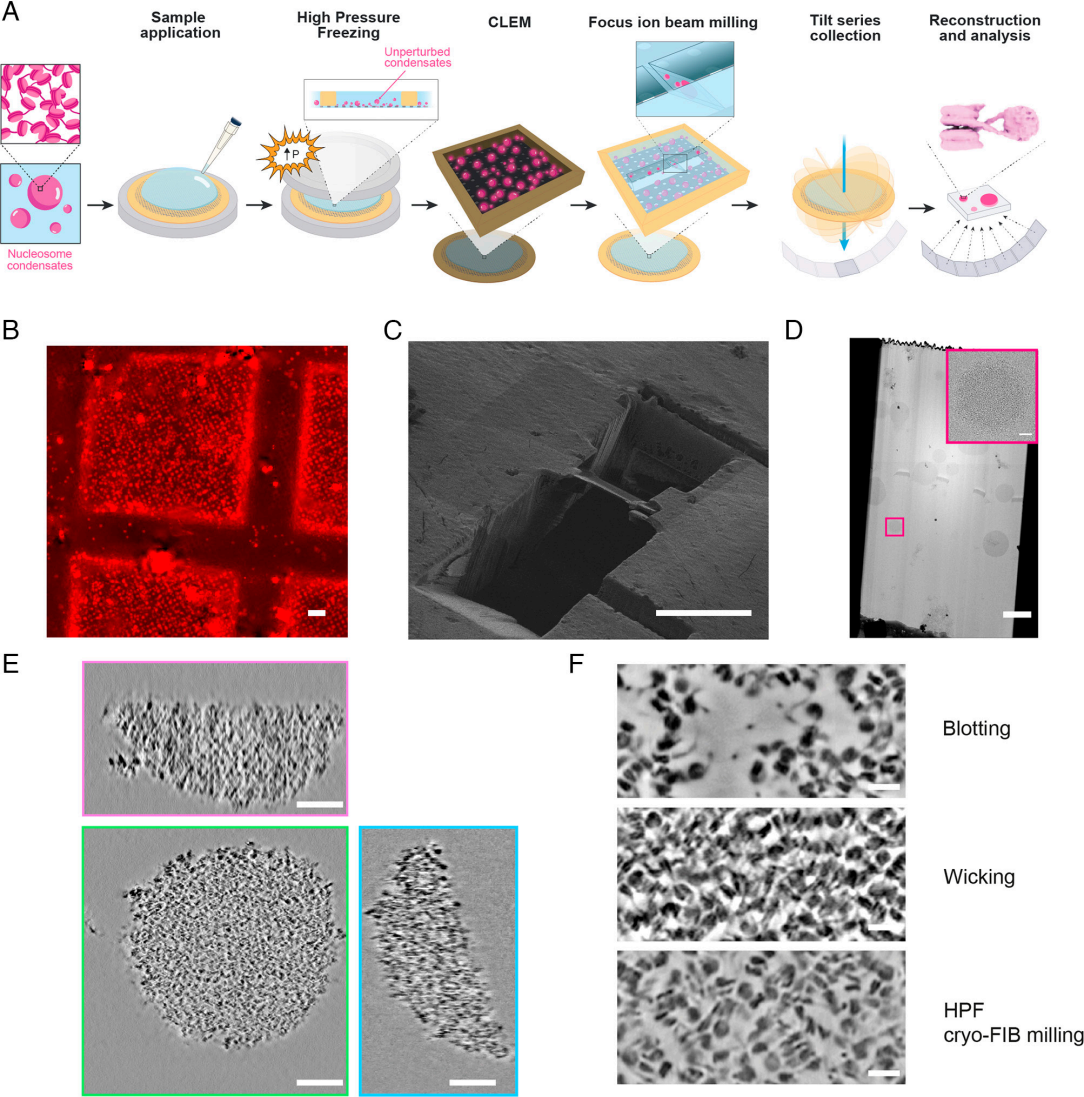
Preservation of droplet morphology via HPF and cryofocused ion beam milling. (*A*) Diagram depicting the integrated approach employing HPF, correlative light and electron microscopy, and cryoelectron tomography to visualize chromatin droplets in their native state. Large condensates cut into discs, and small condensates remain fully round. (*B*) Representative squares of cryofluorescent images. Chromatin was labeled on histone H2B with AF594. (Scale bar, 20 μm.) (*C*) A lamellum produced by FIB-milling. (Scale bar, 20 μm.) (*D*) Representative images of medium magnification montage of a lamellum using cryo-TEM. (Scale bar, 2 μm.) *Inset* is an enlargement of the boxed region. (Scale bar, 200 nm.) (*E*) Orthogonal tomographic cross-sections of chromatin condensates prepared via HPF. (Scale bar, 100 nm.) X-Y, X-Z, and Y-Z views are shown with green, red, and blue outlines, respectively. (*F*) Tomographic sections illustrating chromatin in condensate samples prepared using the indicated methods. (Scale bar, 20 nm.)

To best preserve condensates in their native state and during sample preparation, we turned to high-pressure freezing (HPF) and cryofocused ion beam (Cryo-FIB) milling using the Waffle Method (40). Condensate-containing samples were transferred to the back side of holey carbon grids, and droplets (~0.5 to 3 µm in diameter) were allowed to settle on the carbon film for 30 s before freezing to enrich droplets on the front side of the grid. After HPF, such droplets were fully embedded in the 25 µm thick slab of vitreous ice on the grid (Fig. 2*A*).

We then used cryofluorescence microscopy of the frozen grids with Alexa Fluor 594 (AF594)-labeled chromatin condensates to identify grid squares, and subregions within them, with highly abundant droplets in order to increase the probability of capturing condensates in the milled lamella (Fig. 2*B*). The grids were transferred to the chamber of the Cryo-FIB instrument and the droplet-enriched regions were milled at random sublocations (Fig. 2*C*). Droplets within the lamella were identified via transmission electron microscopy (Fig. 2*D*). Effective template matching (see below) required lamella with 80 to 150 nm thickness. This thickness was important since thicker samples resulted in increased electron scattering, leading to lower resolution and poor contrast, which compromised the quality of the acquired images and hindered accurate structural analysis (41). Using HPF and cryo-FIB milling (Movie S1), we could solve many of the problems associated with blotting and self-wicking methods. HPF produced spherical droplets with little apparent distortion (Fig. 2*E*). These could be generated from condensates that were free-floating in solution and not attached to the carbon supports of the grid. Internally, nucleosomes were relatively uniform in density and the droplets lacked large cavities. No naked DNA could be observed, indicating that the nucleosomes were generally intact. Finally, the overall density of HPF condensates was intermediate between blotted samples, where disruption generally spread nucleosomes, and wicked samples, where dehydration increased nucleosome packing (Fig. 2*F*).

### Assigning Nucleosomes in Synthetic Tomograms with CATM.

**Fig. 3.**
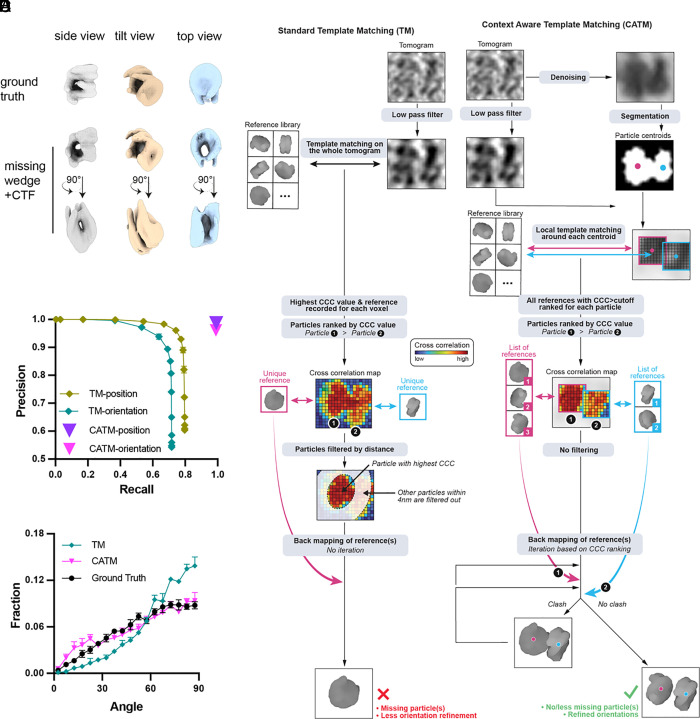
CATM for precise nucleosome localization and orientation in simulated data. (*A*) Simulation of missing-wedge and CTF modulation effects for a pair of stacked nucleosomes from different perspectives. (*B*) Schematic overview of standard template matching algorithm. Each representative voxel only retains the reference image (and corresponding template) with the highest CCC to data, and the particles are then filtered by a distance cutoff between their centers of mass. (*C*) Schematic overview of CATM. Centroids of the models are first identified with deep learning segmentation and localization. The localized particles are then used for local template matching and multiple templates are systematically used for optimization in the assignments. (*D*) Precision and recall for nucleosome assignments (center of mass position) by CATM and TM. The TM algorithm has different performance characteristics for different cross-correlation cutoffs; the cutoff with the highest F1 score was used for later analysis. CATM produces only a single set of assignments. The results are presented as the mean and SD calculated from three tomograms. Note that error bars for CATM are smaller than the symbols. (*E*) Distribution of angle between the normal to the nucleosome plane and the beam direction (Z-axis) as determined by CATM and TM. Nucleosomes are oriented randomly in the ground truth (black circles), producing a sinusoidal distribution. The results are reported as the mean and SD derived from three tomograms.

Understanding molecular interactions and higher-order organization within condensates requires accurate assignments of molecular identity with high coverage in tomograms. This is a challenging task due to the crowded environment inside condensates and technical issues including the “missing wedge” problem and contrast transfer function (CTF) modulation, which render molecular identities ambiguous even for well-characterized structures (42) (Fig. 3*A* and *SI Appendix*, Fig. S3). To evaluate the efficacy of various template matching procedures in identifying individual nucleosomes in condensates, we first simulated tomograms with a mix of nucleosomes and DNA, mirroring the density of the chromatin condensates and the noise level (43) in our images (*SI Appendix*, Fig. S4*A*). Using standard template matching techniques (TM) (44) (*SI Appendix*, *Methods*) applied to these simulated data (Fig. 3*C*), we positioned the center of mass of the template at each voxel, rotated it at various angles, applied the missing wedge, and performed cross-correlation. The highest cross-correlation coefficient (CCC) values then determined the center of mass voxel and orientation of each matched template. In refinement, when two particles had centers of mass within a specified distance cutoff (4 nm, slightly smaller than the width of nucleosome), the one with a lower CCC value was removed. Using various CCC cutoffs we calculated established metrics of machine learning (45, 46), namely recall, precision, and F1 (the harmonic mean of precision and recall; see *SI Appendix*, *Methods*), to compare assigned positions/orientations with the ground truth. Analyzing the nucleosome centers of mass, we obtained a maximum F1 score of 0.85. Adding orientation angles to the comparison reduced the maximum F1 score to 0.76 (*SI Appendix*, Fig. S4 *B*–*F*). Using the well-established template matching program PyTom (44) with default parameters yielded similar results (*SI Appendix*, Fig. S4 *D*–*G*).

The high density of particles in condensates often results in overlap of initially assigned positions during template matching, a problem that is exacerbated by artifacts from the missing wedge and CTF (42). To solve this problem, it is essential to simultaneously optimize particle positions and orientations. Moreover, the nonspherical shape of the nucleosomes necessitates that the assignment process incorporates steric information instead of merely relying on a single center-to-center distance cutoff (47). The template matching struggles in this regard because it treats each voxel uniformly, resulting in an unfeasibly large search space when optimizing orientations of multiple particles simultaneously. To address this complexity, we divided the task into two distinct stages: localization and angular assignment (Fig. 3*C*). Initial localization of the particles was achieved using deep learning–based image segmentation (48). This was followed by refinement of particle orientations and positions through local template matching and pose optimization, taking into account the steric properties of the templates (Fig. 3*C*). Local template matching limited by the image segmentation has a vastly limited search space, allowing simultaneous pair-wise optimization of particle position/orientation.

In practice, we used DeepFinder (48) to segment the denoised tomograms (see below) and determine an initial estimate of the center of mass of each particle. We then use these particle positions as a starting point for a process we term CATM (Fig. 3*C* and Movie S2). During CATM, subtomograms centered at the particle positions were extracted from the low-pass filtered raw tomogram, and the template was rotated at various orientations to calculate the cross-correlation. We retained the orientation with the highest CCC along with those with lower values for each particle (above a threshold of ~0.3, Fig. 3*C*). The mapping process involved iterating through each particle, in descending order of CCC, and positioning them back into a tomogram. If a given particle had a steric clash with previously placed particles, the alternative orientations saved for that particle were examined to resolve the clash. If the clash remained unresolved by these configurations, we paired the nearest neighboring particle with the current particle and tested combinations of orientations for both to settle the conflict. If sterically compatible solutions were found, the orientation pairs that maximized the CCC values were retained and placed in the tomogram. If a resolution was unattainable, only the particle with the higher CCC was saved (*SI Appendix*, Fig. S5).

This method significantly improved the accuracy of nucleosome identification and orientation assignments, achieving both high precision and recall (Fig. 3*D*). The F1 score of 0.99 for position assignment and 0.96 for angular assignment (*SI Appendix*, Fig. S4 *E* and *F*) substantially surpasses the TM performance. Furthermore, an important measure of accuracy in the assigned nucleosome orientations is the distribution of angles with respect to the imaging axes. Since nucleosomes should be randomly oriented both in the simulated data and in real data on frozen condensates (which are isotropic), the distribution of nucleosome planes should be random, i.e. sinusoidal in angular coordinates (49) (Fig. 3*E*, black line). Missing wedge distortions make correct assignment in the Z-dimension particularly difficult. As shown in *SI Appendix*, Fig. S4 *G* and *H*, standard template matching undercounts nucleosomes viewed face-on (low angles between the vector normal to the nucleosome plane and the imaging axis vector, along Z) and overcounts side-on views (high angles between these vectors). In contrast, CATM achieved a random distribution of nucleosome orientations that mirrored the ground truth. Thus, image segmentation followed by CATM enables nucleosomes to be accurately positioned and oriented in simulated tomograms.

### Application of CATM to Chromatin Condensates.

We next sought to apply CATM to reconstructed tomograms generated from tilt series acquired on cryo-FIB milled lamella containing chromatin condensates. To enhance the visualization and segmentation of nucleosomes, we first used Warp (50) and IsoNet (51) to denoise the tomograms (*SI Appendix*, Fig. S6). We manually segmented a small training set volume in DeepFinder and used the assignments to train the neural network for segmentation. We employed the MeanShift (52) algorithm to retrieve the centroids of the particles and to remove particles too close to the surfaces (<20 nm) of the FIB-milled lamella or located external to the condensates (*SI Appendix*, Fig. S5).

Based on the particle locations, we used CATM to assign nucleosome orientations. Both position and orientation were then further refined using Relion (53). We noticed that since particles are independently processed in Relion, refinement has a propensity to cause nearby particles to become overlaid at a single position. This occurs for ~5% to 20% of particles depending on the nature of the condensate (*SI Appendix*, Fig. S7 *A*–*C*). To ensure accurate placement of the maximum number of particles, we merge the results from CATM and Relion, excluding overlaid particles that lead to clashes and adding the missing particles from the CATM result, to achieve a refined set of nucleosome assignments (*SI Appendix*, Fig. S7 *A* and *B*).

**Fig. 4.**
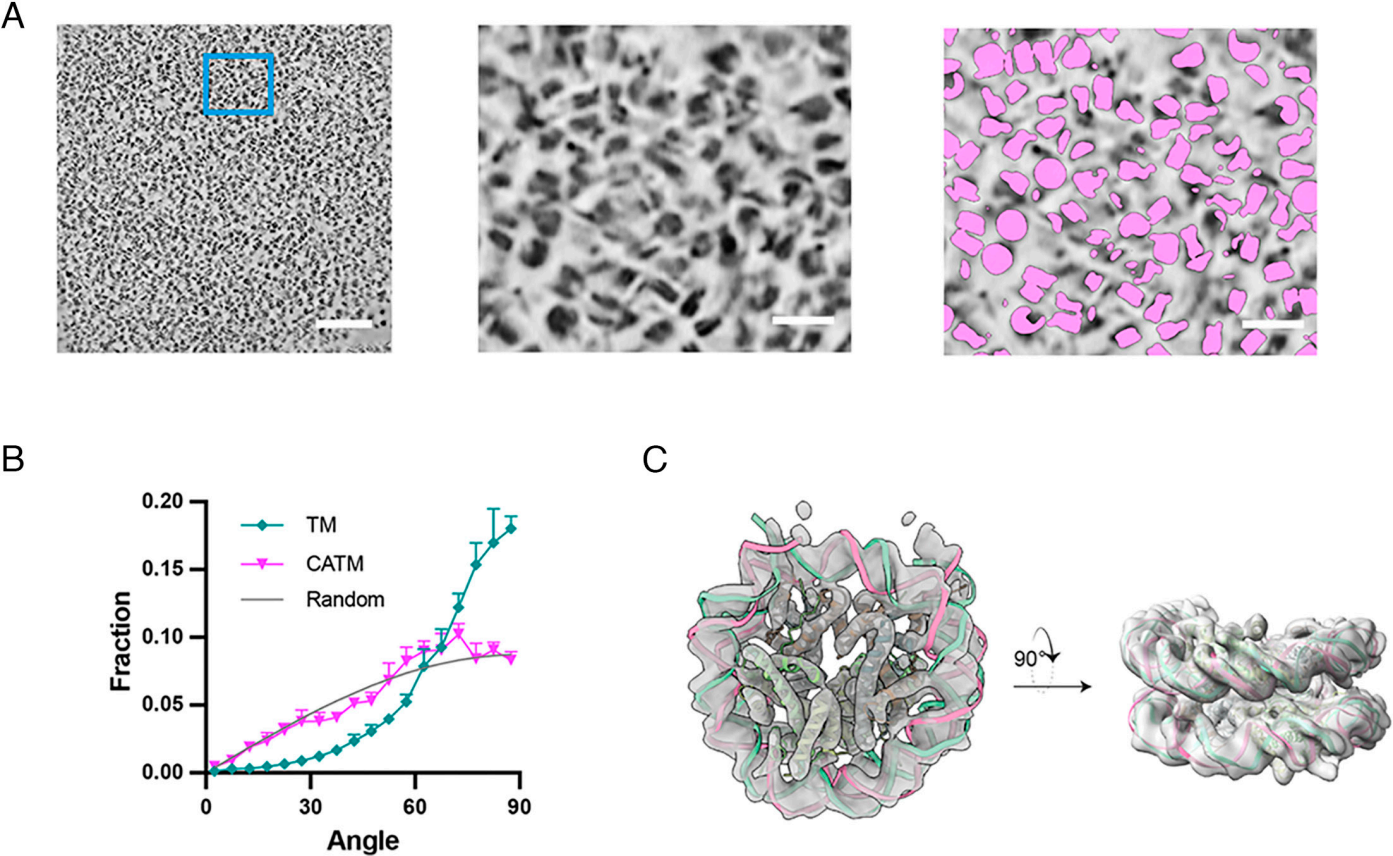
Cryo-ET analysis of reconstituted chromatin condensates. (*A*) Tomographic visualization and CATM analysis of chromatin condensates. The *Left* panel presents a tomographic section through a chromatin condensate. (Scale bar, 100 nm.) The *Central* panel shows an enlarged view of the area delineated by the blue square in the *Left* image. (Scale bar, 20 nm.) The *Right* panel shows CATM nucleosome assignments for the tomographic section in the *Central* panel. (*B*) Distribution of angle between the normal to the nucleosome plane and the beam direction (Z-axis) as determined by the TM (our implementation) and CATM. Random orientations (gray) are sinusoidally distributed. (*C*) Subtomogram averaging of 126,125 particles from 14 tomograms, identified by CATM, aligned with a cryo-EM derived core nucleosome structure (PDB ID: 6pwe).

Visual inspection of the final set of assigned particles mapped back to the tomograms suggested that most nucleosomes were correctly positioned and oriented by the procedures (Fig. 4*A*). Moreover, the distribution of nucleosome orientations was nearly random in the imaging directions, as expected for an isotropic condensate, supporting accuracy of the assignments (Fig. 4*B*). Finally, we extracted subtomograms and performed subtomogram averaging using Relion, which yielded a nucleosome structure at 6.1 Å resolution without discarding any particles (126,126 particles from 14 tomograms acquired from eight lamellae, Fig. 4*C* and *SI Appendix*, Fig. S8 and Table S1). These qualitative and quantitative measures strongly suggest that our image analysis procedures accurately identify the positions and orientations of most nucleosomes within the dense interior of chromatin condensates.

### Nucleosomes are Organized Heterogeneously Within Condensates.

**Fig. 5.**
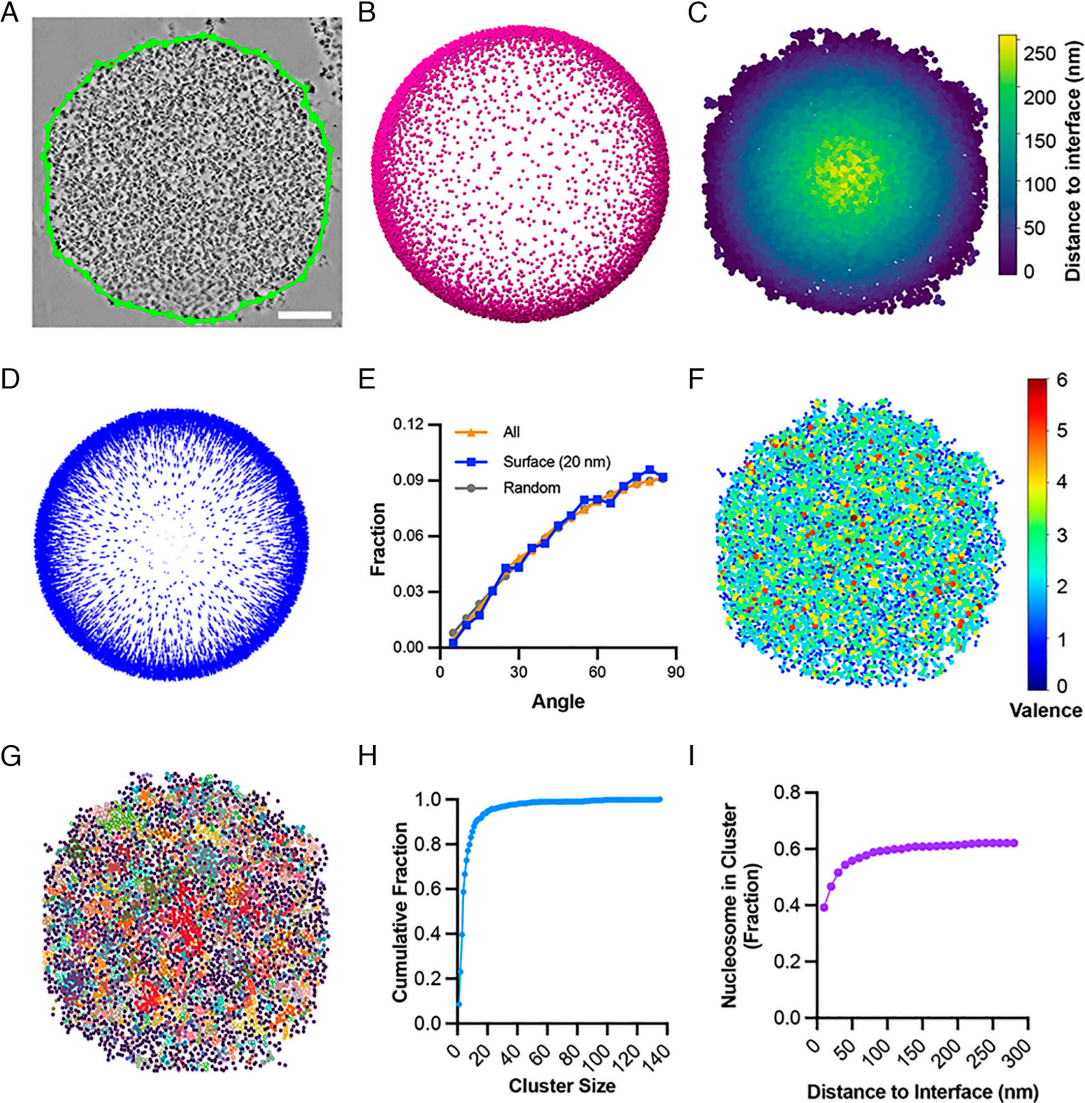
Spatial organization of nucleosomes in the chromatin condensate. (*A*) Manually delineated condensate–buffer interface (green line) on an individual slice of the tomogram. (Scale bar, 100 nm.) (*B*) Fit of the annotated droplet interfaces from each section of the tomogram to a sphere. Surface points that are closest to at least one nucleosome in the lamellum are indicated. (*C*) Heatmap of nucleosome positions (dots), color-coded by their shortest distance to the condensate interface. (*D*) Blue arrows indicating the surface normals of the condensate. (*E*) Distribution of the angle between the nucleosome plane normal and its nearest condensate–buffer interface normal. Data include nucleosomes from five tomograms: the yellow line represents the frequency distribution of all nucleosomes (n = 27,259), while the blue line represents nucleosomes within 20 nm of the condensate interface (n = 2,565). (*F*) Chromatin condensate network graph, where each node represents a nucleosome (not to scale) from panel (*A*), color-coded by the valence of interactions. (*G*) Nucleosome clusters within the condensate, with different clusters shown in distinct colors. Black dots represent nucleosomes not part of any cluster. (*H*) Cumulative distribution of cluster sizes (number of nucleosomes) from panel (*G*). (*I*) Fraction of nucleosomes in clusters as a function of their distance from the condensate interface, based on panels (*C*) and (*G*).

To understand nucleosome organization across the condensate, we initially analyzed nucleosome orientations relative to the condensate–buffer interface to learn whether surface tension might influence their distribution. We manually delineated the condensate perimeter within individual tomographic slices (Fig. 5*A* and *SI Appendix*, Fig. S9*A*), and used the perimeters from all slices in the lamella to fit the surface of the condensate sphere (Fig. 5*B* and *SI Appendix*, Fig. S9*B*). This allowed us to determine the nearest surface position for each nucleosome (Fig. 5 *B* and *C* and *SI Appendix*, Fig. S9 *C* and *D*) and the surface normal vector at that position (Fig. 5*D* and *SI Appendix*, Fig. S9*E*). We then computed the angle between the nucleosome normal and its nearest condensate surface normal (Fig. 5*E*). Our analysis revealed that neither bulk nucleosomes nor those near the interface exhibit a preferred orientation relative to the condensate surface (Fig. 5*E*).

To further understand the nucleosome interactions as a network, we constructed graph networks (Fig. 5*F* and *SI Appendix*, Fig. S9*F*). To assess the heterogeneity of the node valence distribution of the network, we computed the normalized entropy of the distribution (54), where a value of 1 indicates a uniform population of each valence and a value of 0 indicates only one valence is populated. The chromatin network exhibits heterogeneity, with a normalized entropy of valence distribution of 0.74 ± 0.02. This analysis reveals that at high-resolution (10 to 100 nm) nucleosomes in the condensates are organized heterogeneously, with small clusters of high density and locally high valence surrounded by regions of low density/valence. Such mesoscale heterogeneity has been observed in computational (55) and experimental (56) studies of condensates produced by intrinsically disordered proteins, suggesting that it may be a general feature of phase-separated compartments.

We further investigated the clustering of high-valency nucleosomes within the condensate using DBSCAN (density-based spatial clustering of applications with noise) (Fig. 5*G* and *SI Appendix*, Fig. S9*G*). This algorithm identifies clusters by analyzing the local neighborhood of each nucleosome, grouping densely connected ones while treating sparsely distributed nucleosomes as noise. Our analysis revealed that most clusters contain fewer than 20 nucleosomes, with a peak at four nucleosomes, although the distribution exhibits a long tail (Fig. 5*H* and *SI Appendix*, Fig. S9*H*).

Finally, we examined the fraction of nucleosomes within clusters across the condensate and found that nucleosomes at the interface are less likely to associate with clusters (Fig. 5*I* and *SI Appendix*, Fig. S9*I*). This finding provides insight into the potential origins of condensate surface tension: At the interface, nucleosome–nucleosome interactions are less saturated and experience an imbalance of forces, making them more likely to be pulled inward. This effect mirrors classical surface tension in liquids, where molecules at the boundary experience asymmetric interactions, driving condensation and minimizing surface area.

Thus, our pipeline from sample preparation to image analysis has enabled us to visualize the internal structure of in vitro reconstituted chromatin condensates, from individual nucleosome structures to the mesoscale organization of the interaction network.

### Application of CATM to In Situ Native Chromatin.

We next sought to apply our methods to native chromatin, which is appreciably more complicated than our synthetic condensates. Complexity arises because in the cell, nucleosomes are composed of different histone variants (57), are covalently modified with diverse epigenetic marks (58), are separated by DNA linkers of variable length (59), and are bound to other macromolecular components (60). Nevertheless, the relatively stereotypical structure of the nucleosome, coupled with previous cryo-ET applications to native chromatin (30–34), suggested our approaches could be effective even in this more complicated system.

**Fig. 6.**
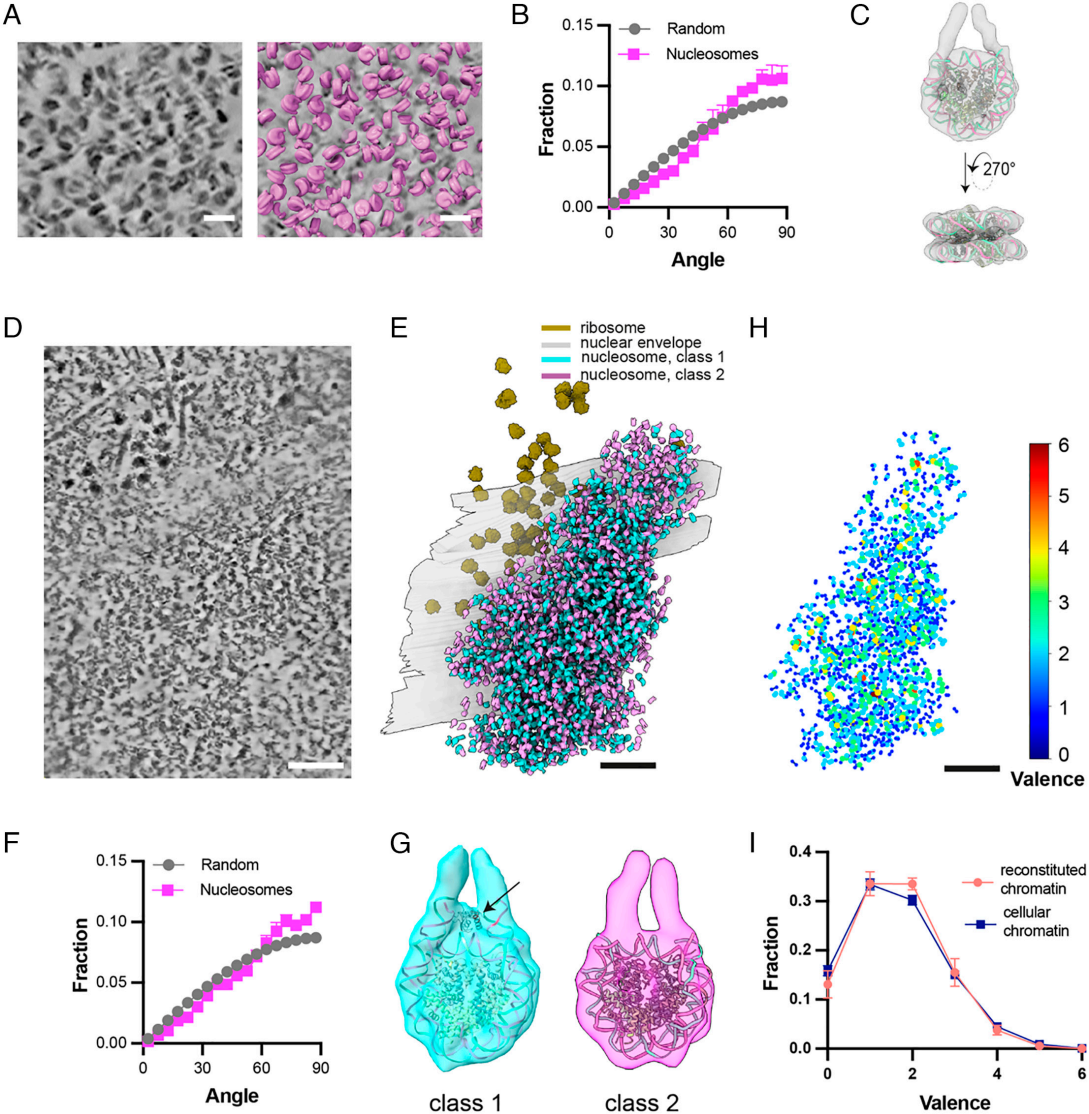
Configuration of native chromatin in isolated HeLa nuclei and intact NIH3T3 Cells. (*A*) A tomographic slice through purified HeLa cell nuclei (*Left*) with superimposed nucleosome models assigned by CATM (*Right*). (Scale bar, 20 nm.) (*B*) Distribution of angle between the normal to the nucleosome plane and the beam direction (Z-axis) as determined by CATM of the 35,503 nucleosomes identified within 13 tomograms from three purified HeLa cell nuclei. (*C*) Subtomogram average of nucleosome structure extracted from the HeLa cell nuclei with resolution is 12 Å, aligning with nucleosome structure (PDB ID: 6pwe). (*D*) Cross-section from a denoised tomographic volume of an NIH3T3 cell. (Scale bar, 100 nm.) (*E*) Segmented visualization of the nuclear envelope, ribosome, and nucleosomes from the tomogram in (*D*) but with whole tomogram annotation, illustrating nucleosomes assigned by CATM (magenta and cyan), ribosomes (brown), and the nuclear envelope (gray) embedded within the volume. (*F*) Distribution of angle between the normal to the nucleosome plane and the beam direction (Z-axis) as determined by CATM of 13,274 nucleosomes from four tomograms of NIH3T3 cells. (*G*) Subtomogram average depicting the two classes of nucleosome reconstructed from a total of 6,470 and 6,804 particles in four tomograms from NIH3T3 cells. The resolution is 12 Å for class 1 and 22 Å for class 2, with the maps fitted to nucleosome structures (PDB IDs: 6pwe and 4qlc, respectively). The black arrow points to probable linker histone density at the nucleosome dyad. (*H*) Graph network of the cellular chromatin condensate, where each node represents a nucleosome (not to scale) from panel (*E*) and is color-coded based on the valence of interactions it mediates. (*I*) The valence distribution of chromatin graph networks in both reconstituted chromatin (illustrated in Fig. 4) and cellular chromatin from NIH3T3 cells, presented as the mean and SD derived from four tomograms.

We purified HeLa cell nuclei and prepared lamella using HPF and cryo-FIB milling. Consistent with superresolution imaging of nuclear DNA (61, 62), native chromatin shows numerous 100 to 300 nm diameter regions of high signal separated by regions of low signal. Within the former, numerous nucleosomes can be observed readily by inspection (Fig. 6*A*). We focused our analyses on these nucleosome-rich regions. These samples yielded high-quality tomographic data, enabling us to precisely map nucleosomes using CATM (Fig. 6*A*). In the absence of specific landmarks that might locally influence the organization of chromatin, we would expect nucleosomes to be randomly oriented, as in our in vitro condensates. Consistent with this idea, nucleosome orientations in native chromatin were predominantly random with respect to the imaging axes (Fig. 6*B*). This random distribution supports both the maintenance of sample integrity during freezing and the efficacy of our algorithm. Subsequent subtomogram averaging yielded a nucleosome structure with a resolution of 12 Å, based on 35,503 particles observed in three lamellae (Fig. 6*C* and *SI Appendix*, Fig. S10*A* and Table S1).

We further extended our approaches to plunge-frozen NIH3T3 cells, where we collected tilt-series on cryo-FIB milled lamella containing nuclei. Within the peripheral regions of these nuclei, we identified various cellular components, including ribosomes, the nuclear envelope, and nucleosomes. Postdenoising, regions containing nucleosomes at high density were clearly distinguishable (Fig. 6*D* and *SI Appendix*, Fig. S10*B*). Within these regions, individual nucleosomes could be assigned using CATM (Fig. 6*E* and *SI Appendix*, Fig. S10 *C* and *D*). The near-random distribution of nucleosome orientations relative to the imaging axis confirmed the accuracy of the algorithm and indicates that in vivo the higher-order organization of chromatin may not be strongly influenced by neighboring cellular structures (Fig. 6*F*). Subtomogram averaging yielded two different classes of nucleosome structures, with ~12 Å and 22 Å resolution, respectively (Fig. 6*G* and *SI Appendix*, Fig. S10*E* and Table S1). Notably, in the former (Class 1), the reconstructed structure included density consistent with linker histone, which binds to the nucleosome dyad axis with high stoichiometry in mammalian cells (63) (Fig. 6*G*). The ratio of nucleosomes in Class 1 and Class 2 is 48.7%:51.3%. To investigate potential spatial relationships between these structures, we constructed a graph network and analyzed the attribute assortativity coefficient (ASC) of the two classes. ASC quantifies the tendency of nodes with similar attributes to connect, ranging from −1 (indicating minimal connectivity between similar nodes) to +1 (indicating strong preferential connectivity). Our analysis yielded a near-neutral ASC value of 0.03 ± 0.02, suggesting random mixing between the two classes. Additionally, the ratio of linker histone H1 to nucleosomal core particles in mouse cells varies between 0.5 and 0.8, depending on the cell type (64). Together, these results suggest that the two observed classes may represent nucleosomes bound and unbound to linker histone, and if so, that linker histone binds randomly to the nucleosomes in our samples.

In contrast to the reconstituted chromatin condensates, where the entry and exit DNA of nucleosomes was not visible in reconstructions (Fig. 4*C*), in both of the cellular samples this DNA is readily observed (Fig. 6 *C* and *G*), suggesting less conformational variability. The more limited conformational space in cells could arise from several factors including DNA linker lengths, nucleosome binding molecules, different histone variants, or histone posttranslational modifications and/or their reader proteins. Further studies correlating nucleosome organization and structure with these factors will be necessary to understand these observations.

Finally, constructing graph networks of the cellular chromatin revealed that like the reconstituted condensates, nucleosomes are organized heterogeneously with normalized entropy of valence distribution of 0.77 ± 0.01 (vs. 0.74 ± 0.02 for reconstituted chromatin condensates, see above and Fig. 5*F*). Small foci of high local density and valence are interspersed within larger regions where both parameters are lower (Fig. 6*H*). The similarity of the network valence heterogeneity and distribution (Fig. 6*I*) found in native chromatin and synthetic condensates suggests that cellular chromatin fibers, despite their vastly longer lengths, have sufficient plasticity to pack analogously to much smaller fragments. The similar packing in cells and in reconstitutions likely arises from the relatively stereotypical structure of nucleosomes and the rigidity of linker DNA. These characteristics of chromatin may be analogous to the fact that within folded proteins, amino acids adopt packing and geometries that are similar to those found in amino acid crystals (65).

## Discussion

There is growing recognition that biomolecular condensates have highly complex physical properties (66). Most behave as heterogeneous network fluids, with internal substructures on different length scales (67), as well as differences in molecular organization between their cores and surfaces (68, 69). Moreover, their internal solution environments can differ substantially from the surrounding media, for example in pH, salt concentration (70, 71), and hydrophobicity (72). To fully understand how these properties affect chemistry within condensates, it is necessary to understand the structures of individual components as well as their higher-order organization.

Our results demonstrate the effectiveness of HPF and focused ion beam (FIB) milling [the waffle method (40)], coupled with cryo-ET and image analysis, to achieve this goal. The pipeline we have developed here overcomes two substantial obstacles in studies of condensate structure. First, due to the liquid-like properties of condensates, inappropriate preparation of biochemically reconstituted samples can lead to artifactual distortions of both their overall architecture (Fig. 1*B*) and their internal packing arrangements (Fig. 1*E* and *SI Appendix*, Fig. S1*F* and S2*B*). By contrast, HPF is highly structure-preserving. Second, the high density of condensates, both biochemically reconstituted and cellular, can lead to inaccurate molecular assignments, introducing artifacts such as apparent preferred orientation (Figs. 3 and 4*B* and *SI Appendix*, Fig. S4 *D–H*), which impair interpretation of higher-order organization and can degrade the quality of subtomogram analysis. Our CATM algorithm utilizes both features of the images and prior knowledge of molecular structure to achieve accurate assignments. We note that while artifacts of sample preparation and image analysis are readily identifiable in chromatin condensates due to the nature of nucleosomes (large, stereotypical structure; high electron density), they may not be as obvious in other types of condensates. Nevertheless, they are expected to occur in cryo-ET studies of many liquid-like systems of high density, emphasizing the importance of the workflow we have developed here. These methods now open the door to understanding how the internal structure of biomolecular condensates influences diverse processes.

Further technical improvements should advance the capabilities of our pipeline. Recently matured plasma-FIB milling (73) will enhance the efficiency of sample preparation, as it can process thick ice layers more rapidly and potentially yield more stable lamellae. Additionally, for condensates typically less than 10 µm in diameter, employing specially designed grids with thinner bars could reduce stress on the samples during milling, leading to faster processing and more stable lamellae. Moreover, advanced laser phase plate technology (74) holds great promise for visualizing and analyzing smaller, more heterogeneous samples in both in vitro and in vivo settings. Our CATM algorithm may be enhanced by incorporating GPU acceleration, which could improve angular sampling during template matching, thereby refining the precision of angular assignments. Moreover, expanding from two-body to multibody optimization, possibly incorporating Monte Carlo sampling, could provide even more accurate assignments, advancing our understanding of complex interactions within condensates.

Human cells compact 2 m of DNA into a 10 µm nucleus by wrapping DNA around histones to form nucleosomes, which assemble into higher-order structures and interact with proteins to regulate gene transcription and cell fate. A key question is how the structures, interactions, and 3D organization of chromatin regulate cellular processes across multiple scales. Studies have revealed that cellular chromatin forms small clutches (~10 to 50 nucleosomes) (75, 76) and larger Topologically Associated Domains (TADs, ~1,000 to 5,000 nucleosomes), but the precise positions and orientations of nucleosomes within these assemblies remain elusive due to technical challenges (76, 77). While selective DNA labeling improves visualization of chromatin at finer scales (78), it often sacrifices structural details. Cryo-EM offers great potential to observe chromatin in its native state, particularly when samples are generated with HPF combined with cryo-FIB milling (34, 40, 79). Reliable preparation of native samples, coupled with advanced quantitative analysis, could enable precise probing of chromatin network interactions and their functional implications. Furthermore, advanced algorithms capable of automatically tracing nucleosome chains in chromatin could provide insight not only into the conformations of small clutches but also the connectivity within TAD-like domains.

Most of the cryo-ET analyses described here focused on biochemical reconstitutions, where knowledge of component parts greatly simplifies template matching. Nevertheless, we have shown that our pipeline can be extended to certain cellular structures where selected components are highly enriched and have stereotypical structures, as in chromatin studied in nuclei here. Other cases may include condensates containing recognizable filaments, as in centrosomes (80) or immune signaling condensates (10), or large machines, as in transcriptional (81) or splicing foci (82). Advances in correlative cryo-super-resolution light microscopy and cryo-ET may enable molecular-scale structural analyses of other condensates as well (83). Ultimately, the combination of biochemical reconstitutions of increasing complexity (7, 10, 80) plus in situ analysis of cellular condensates is likely to provide the greatest depth of mechanistic understanding.

In conclusion, by ensuring the integrity of reconstituted condensates and accurate molecular assignments, our study establishes a workflow for structural analysis of both biochemically reconstituted and native chromatin condensates, which should be applicable to certain other condensates as well. Using it, we have been able to determine structures of nucleosomes in both reconstituted chromatin condensates and native chromatin. Additionally, we found that higher-order nucleosome packing is similarly heterogeneous in both cases. Future studies will address key issues in the condensate and chromatin fields, including mechanisms of phase separation, the activities of resident molecules, and the influence of condensate environment on internal chemistry.

## Materials and Methods

Detailed methods for the assembly of nucleosome arrays, preparation of cryo-EM grids, cryo-FIB milling, and cryo-ET data analysis are provided in *SI Appendix*.

## Supplementary Material

Appendix 01 (PDF)

Movie S1. To preserve chromatin condensates in their native state, we used highpressure freezing and cryo-FIB milling (the Waffle method) to prepare lamellae. The chromatin droplet solution was applied to the back side of the cryo-EM grid and rapidly vitrified using a highpressure freezer. The grid was then imaged with a cryo-fluorescence microscope to localize the chromatin condensates. Subsequently, the grid was transferred to a focused ion beam scanning electron microscope (cryo-FIB-SEM) to remove excess material and produce thin lamellae. After bulk milling, notch milling was performed to ensure lamellae stability. Thin lamellae (<150 nm) were critical for achieving high image quality. The prepared lamellae were then transferred to a cryo-electron microscope, where tilt-series were collected using a dose-symmetric scheme. Finally, the tomograms were reconstructed via back-projection, and nucleosomes were properly assigned using our algorithm.

Movie S2. In the crowded condensate environment, molecules are densely packed, making it challenging to determine the exact number of nucleosomes, as seen with two stacked nucleosomes in this example. To address this, we tilt the sample and collect tilt-series at various angles. However, due to stage limitations, some angles cannot be sampled, resulting in a missing wedge of information. Combined with contrast transfer function (CTF) modulation, this distortion elongates particles along the beam direction, complicating their identification. Here, we compare standard template matching (TM) with our context-aware template matching (CATM). In TM, the program searches for the best fit at local minima, excluding particles based on distance metrics, which often leads to errors in particle number, orientation, or both. In contrast, CATM uses a more robust pipeline: first, particle centroids are identified using deep learning-based segmentation or picking. Local template matching is then performed around these centroids, recording multiple orientation and position possibilities for each particle. Finally, particles are mapped back into the tomogram, and potential clashes are resolved by optimizing their orientation combinations. This approach significantly improves accuracy in identifying both the number and orientation of particles.

## Data Availability

The TM and CATM software and all analysis scripts are available on GitLab (https://git.biohpc.swmed.edu/rosen-lab/catm) (84). The cryo-ET maps have been deposited in the EMDB under the following accession codes: nucleosome from reconstituted chromatin (Fig. 4*C*)—EMD-49924 (85); nucleosome from HeLa nuclei (Fig. 6*C*)—EMD-49923 (86); nucleosome from NIH3T3 (Fig. 6*G*)—EMD-53275 (class 1) (87) and EMD-49929 (class 2) (88).
