# Supplementary material for: Quantitative spatial analysis of chromatin biomolecular condensates using cryoelectron tomography
Source: Proc Natl Acad Sci U S A. 2025 May 6;122(19):e2426449122. doi: 10.1073/pnas.2426449122 (PMC12088439; doi:10.1073/pnas.2426449122)
Supplement: Supplementary file 1 — Appendix 01 (PDF) [file pnas.2426449122.sapp.pdf]

PNAS

Supporting Information for

Quantitative spatial analysis of chromatin biomolecular condensates using cryoelectron tomography

Huabin Zhou[a], Joshua Hutchings[b], Momoko Shiozaki[c], Xiaowei Zhao[c], Lynda K. Doolittle[a], Shixin Yang[c], Rui Yan[c], Nikki Jean[c], Margot Riggi[d], Zhiheng Yu[c], Elizabeth Villa[b,e], Michael K. Rosen[a]
[a]Department of Biophysics, Howard Hughes Medical Institute, UT Southwestern Medical Center, Dallas, TX, 75390, USA
[b]School of Biological Sciences, University of California San Diego, La Jolla, CA 92093
[c]Janelia Research Campus, Howard Hughes Medical Institute, Ashburn, VA, USA 20147
[d]Research Department Cell and Virus Structure, Max Planck Institute for Biochemistry, Martinsried/Munich D-82152, Germany
[e]Howard Hughes Medical Institute, University of California San Diego, La Jolla, CA 92093

Correspondence: michael.rosen@utsouthwestern.edu (M.K.R.), evilla@ucsd.edu (E.V.)

This PDF file includes:

Methods
Figures S1 to S10
Tables S1
Legends for Movies S1 to S2

Other supporting materials for this manuscript include the following:

Movies S1 to S2

Methods

Protein purification and nucleosome array assembly

The nucleosome arrays were constructed as previously described (1). Briefly, human histones H2A, H2B, H3, and H4 were recombinantly expressed in *E. coli*, purified chromatographically and assembled into octamers. Octamers were assembled using salt dialysis onto DNA consisting of 12 repeats of the Widom 601 nucleosome positioning sequence. Labeling was performed by incorporating 1% of AF594-labeled H2BT116C octamers during assembly. A 25% excess of H2A/H2B dimer was added to facilitate complete octamer loading. DNA fragments of ~300 bp were used as carrier DNA to absorb the excess histones to avoid over assembly. The assembled nucleosome arrays were purified using sucrose gradient centrifugation. Sucrose was removed by dialysis and the chromatin was then concentrated by ultrafiltration to 6-8 μM in 20 mM Tris-HCl, pH 7.5, 1 mM EGTA, 1 mM DTT. Array quality was assessed by *Bst* XI into mononucleosomes, followed native polyacrylamide gel electrophoresis (PAGE). Only samples with >90% properly assembled octamer (rather than under-assembled hexamer, lacking one copy each of H2A and H2B) were analyzed further.

Purification of HeLa Cell Nuclei

HeLa cell pellets ($5x10^9$ cells, Ipracell) were resuspended in 5 volumes of lysis buffer (20 mM HEPES, pH 7.9, 5 mM $Mg(OAc)_2$, 0.15 uM Spermine, 10 mM Sodium Butyrate, 10 mM Nicotinamide, 10 uM Pepstatin, 3 mM AEBSF, 10 uM E-64, 2 uM Antipain, 500 uM leupeptin, 2 ug/ml aprotinin, 4 uM TSA, 1 mM DTT), and swelled on ice for 10 minutes. Cells were lysed with 0.05% NP-40 and after gentle inversion were kept on ice for 10 minutes. The lysate was centrifuged at 1000 x g for 10 minutes at 4 °C to pellet the nuclei. The supernatant was carefully removed, and the nuclear pellet was washed twice with lysis buffer containing 0.34 M sucrose by resuspension and subsequent centrifugation at 1000 x g for 10 minutes to remove cytoplasmic contaminants. The integrity and purity of the isolated nuclei were verified by light microscopy. Purified nuclei were flash frozen in liquid nitrogen and stored at -80 °C. For HPF experiments, frozen nuclei were resuspended in freezing buffer (20 mM HEPES, pH 7.9, 100 mM KOAc, 1 mM $Mg(OAc)_2$, 0.15 uM Spermine, 10 mM Sodium Butyrate, 10 mM Nicotinamide, 10 uM Pepstatin, 3 mM AEBSF, 10 uM E-64, 2 uM Antipain, 500 uM leupeptin, 2ug/ml aprotinin, 4 uM TSA, 1 mM DTT).

Grid preparation

<u>Blotting method</u>

For sample preparation using the Vitrobot Mark IV (Thermo Fisher), Lacey carbon grids (200 mesh, EMS) were glow discharged at 30 mA for 30 seconds prior to use. Nucleosome arrays were first equilibrated in a dilute buffer (20 mM Tris-OAc, pH 7.5, 0.1 mM EGTA) at a concentration of 1 μM. They were then mixed 1:1 with buffer containing 40 mM Tris-OAc, 150 mM KOAc, 2 mM $Mg(OAc)_2$, and 0.2 mM EGTA to induce phase separation. The droplets formed instantly and solutions were transferred to grids within 10 minutes. Blotting was conducted at 4 °C and 100% humidity. 3 μL of chromatin solution was applied to the grids, blotted with force 0 for 3.5 seconds, and plunge frozen in liquid ethane, yielding samples with 60 nm to 120 nm thickness.

For single-side blotting, we used an in-house manual plunge freezer. The sample and grids were prepared as described above, with chromatin droplets added to the frontside of the grid. The grid was blotted with filter paper from the backside for 3.5 seconds and then plunge-frozen in liquid ethane.

<u>Self-wicking method</u>

Utilizing the Chameleon system (SPT Labtech) for the self-wicking method, nucleosome arrays were pre-equilibrated in a dilute buffer (20 mM Tris-OAc, pH 7.5, 0.1 mM EGTA) at 0.5 μM concentration, mixed 1:1 with buffer containing 40 mM Tris-OAc, 300 mM KOAc, 2 mM $Mg(OAc)_2$, 0.2 mM EGTA to induce phase separation. Within 10 minutes, 5 μl of the phase-separated chromatin solution was applied to self-wicking grids, which had been glow discharged at 12 mA for 20 seconds using the internal glow discharger. A strip of liquid was sprayed onto the grid, allowed

to wick for 120 milliseconds before plunge freezing, yielding samples with 80 nm to 160 nm thickness.

Waffle method

Prior to sample preparation, planchets for the high-pressure freezer were pre-treated with 0.05% lecithin and Quantifoil carbon 200 mesh EM grids (Quantifoil) were glow discharged at 15 mA for 30 seconds. Lecithin treatment proved less contamination than other solvents (e.g. hexadecane) in affording flat vitreous ice surfaces of the slab following freezing. For chromatin condensates, the nucleosome arrays were pre-equilibrated in a dilute buffer at 6 µM concentration, then mixed with buffer containing 40 mM Tris-OAc, 300 mM KOAc, 2 mM $Mg(OAc)_2$, 10% glycerol, 0.2 mM EGTA to induce phase separation. After placing the first planchet on the HPF tip holder, the EM grid was placed on the planchet with the back side facing up, 4 µL of the phase separated chromatin sample was applied (within 10 minutes of inducing phase separation) to the EM grid, and allowed to sit for 30 seconds before assembly of the HPF tip holder. Settling time was an important optimization parameter to ensure a sufficient density of condensates near the vitreous ice surface to afford efficient targeting during subsequent FIB milling. The sample-loaded grids were inserted into a Wohlwend HPF Compact 03 machine, where rapid freezing was conducted at 2050 bar using liquid nitrogen at -196°C. Following freezing, the grids were disassembled from the HPF tip and stored in liquid nitrogen until required for cryo-fluorescent imaging. For the purified nuclei, the sample was frozen using the same procedure as the reconstituted chromatin.

Plunge freezing of NIH3T3 cells

NIH-3T3 cells (ATCC CRL-1658) were maintained at 37 °C, 5% CO_2 in DMEM (Gibco 11995073). Approximately 1.25×10^5 trypsinized cells were seeded onto glow-discharged EM grids (carbon-coated Quantifoil R1/4 Au200-C) and allowed to adhere for 2 hours at 37 °C, 5% CO_2. Cells were treated with 100 nM jasplakinolide for two hours. Grids were manually blotted with Whatman filter paper #1and plunged in liquid ethane/propane (50:50, AirGas) using a custom-built plunging and vitrification device (Max Planck Institute for Biochemistry, Munich).

Correlative cryo-Light and electron microscopy (CLEM) for condensate samples

Chromatin droplet cryo-grids were examined using a Leica cryo-thunder CLEM system equipped with a 50x/dry objective (NA=0.9). For fluorescence detection, TXR filter cubes (emission wavelength: 592-668 nm) were utilized. The fluorescence intensity manager was set to 100% with an exposure duration of 600 ms. To comprehensively image the entire grid, an 8x8 tile array encompassing a z-stack of 10-20 µm with a 0.5 µm step increment was assembled using Leica LAS X software. These z-stack montages were then processed into 2D CLEM images via maximum intensity projection, facilitating subsequent correlation with cryo-FIB milling processes.

Cryo-FIB-milling

HPF samples (reconstituted chromatin and purified nuclei)

Lamellae were milled using the waffle method (2, 3) followed by manual polishing. Grids were loaded onto a pre-tilted shuttle (45° or 35°), then transferred into an Aquilos 2 cryoFIB-SEM (Thermo Fisher Scientific). The stage temperature was maintained below –180 °C. A grid overview SEM image was taken at 2 kV, 13 pA, then an image of the grid center was acquired with a higher voltage (5-20 kV, 13 pA) to visualize the center landmark of the grid. A CLEM image was imported to Maps (v.3.16-3.25) and then aligned to the SEM image by using the center landmark shape of the grid. Squares with abundant fluorescent droplets were picked and subregions with dense droplets were targeted during the milling. The grids were sputter coated with platinum (30 mA, 10 Pa, 15s) then an organometallic platinum layer was deposited by a gas injection system for 2.5-3 minutes.

Precuts were milled following the waffle method protocol (2, 3) with a beam current of 7-15 nA at 30 kV. Next, a secondary organometallic platinum GIS layer was deposited for 2.5-3 minutes to ensure sufficient GIS layer left through the following procedure. An updated SEM image was taken to highlight the locations of the precuts in Maps software. AutoTEM (v.2.0-2.3, ThermoFisher Scientific) was used to define eucentric position and milling position for each lamella site, then the

underside of the potential lamella was manually milled using a beam current of 3-5 nA at three incrementally decreasing milling angles; 40°, 30°, 20° for a 35° pre-tilted shuttle, or at two angles; ~24°, 20° for a 45° pre-tilted shuttle. SEM images were taken to inspect and monitor the lamella to avoid double layers. At the 20° milling angle, a notch pattern (2, 3) was milled using a beam current of 0.3 nA for 2.5 minutes. AutoTEM was then used to automate the milling at each lamella site, aiming for a final thickness of 180-300 nm at 20° milling angle. The lamella width was set to 12 µm to balance the stability and size of the lamella. The FIB-milling parameters described in the waffle method (2, 3) were used with adjustments of depth correction.

After AutoTEM milling, the lamellae were manually polished to ~100-150 nm, utilizing stage over-tilts of +0.5° (20.5°) and –0.2° (19.8°) from the original milling angle (20°). A rectangle pattern or cleaning cross section (CCS) was used to polish the lamella with a beam current of 10-50 pA. For CCS pattern, Z size was set to 1 µm so the lamella is not damaged during milling. Lastly, the stage was returned to the original milling angle of 20° for a final polish. We note that lamella thickness < 150 nm was essential to achieving high quality cryoET data that were sufficient for accurate template matching subsequently.

Plunge frozen samples (intact NIH3T3 cells)
Grids were milled using an Aquilos 2 cryoFIB-SEM (Thermo Fisher Scientific) using a combination of automated milling (AutoTEM) and manual polishing with 10 pA. Briefly, milling progressed from rough to fine milling using currents at 500, 300, 100, 50, 30 and 10 pA aiming for a nominal lamella thickness of 150 nm. Lamella were polished at 10 pA with 0.5° overtilt for approximately two minutes to remove thicker material from the back of the lamella.

Cryo-ET Data Acquisition
Blotting and self-wicking samples
Data acquisition for samples prepared by blotting and self-wicking was carried out using a Titan Krios G1 (Thermo Fisher Scientific) equipped with a K3 camera (Gatan, Inc) operating at 300 kV. Tilt series were captured ranging from -60° to +60°, with a 3-degree increment per tilt. Images were recorded at a pixel size of 0.206 nm at the specimen level. A Volta phase plate was employed, and the defocus was set to -0.5 µm. To minimize radiation damage, the total electron dose was limited to 150 electrons/Å^2.

Condensate lamellae samples and purified nuclei
For condensate lamellae, data acquisition was conducted using a Titan Krios G3 (Thermo Fisher Scientific) featuring a cold-field emission gun, a Selectris X imaging filter, and a Falcon 4i camera. Tilt series ranged from -48° to +60° with a 2-degree increment per tilt. Each image was captured at a physical pixel size of 0.1516 nm at the specimen level. The defocus ranged between -3 µm and -4.5 µm, with a total electron dose capped at 178 electrons/Å^2.

Cellular samples
Milled grids were imaged on a Titan Krios G3 microscope (Thermo Fisher Scientific) operated at 300 keV equipped with a K3 detector and 1067HD BioContinuum energy filter (Gatan) with 15 eV slit-width. Dose-fractionated images were acquired using SerialEM (4). Dose-symmetric tilt series were acquired in low dose mode using the parallel cryo-electron tomography (PACEtomo) scheme (5) with 3° increments and +/- 54° tilt range from a 6-12° lamella pre-tilt. A total dose of approximately 140 e/Å^2 at 1.34 Å/pixel and 4 µm nominal defocus was used (Supplementary Data Table 1).

Tilt-series Processing and Alignment
The acquired movie frames underwent gain correction, motion correction and Contrast Transfer Function (CTF) estimation using Warp (6). Subsequent tilt series creation and alignment were performed in AreTomo(7). These alignments facilitated further processing in Warp to reconstruct tomograms at 8 Å/pixel. Additionally, two half-sets tomograms were constructed to enable denoising.

Tomogram denoising and segmentation
Initially, the two half-sets of tomograms along with their respective CTF models were used to train a denoising model in Warp, undergoing 40,000 iterations. This process significantly enhanced the contrast of the tomograms. Subsequently, the denoised tomograms were processed through IsoNet for an additional round of missing-wedge restoration and further denoising. The models were specifically retrained for each dataset to ensure optimal performance.

For segmentation, a small section of the tomogram measuring 256x256x304 voxels was manually annotated to identify nucleosome-containing voxels, which served as the training set for DeepFinder. Following training of the neural network, the full tomograms were segmented to identify voxels representing nucleosomes.

The centroid of each particle was then determined using MeanShift, which clustered the segmented maps within a window size of 4.5 pixels. To avoid artifacts from the gallium ions used in the milling process (8), centroids located less than 20 nm from the FIB-milled surface were removed from the dataset. Due to imperfections in the segmentation, regions with a noisy background outside of the condensate sometimes were also picked. We used an intensity threshold to remove such false positive particles.

For native chromatin we used WARP/IsoNet to denoise the tomograms, and DeepFinder/MeanShift to segment the images for nucleosomes as above. Then we used IMOD to manually delineate regions with a high density of nucleosomes, and only these were used for downstream analysis. To annotate nuclear envelopes, segmentation was performed in IMOD and a custom script was used to generate a binary mask. Ribosome positions and orientations were sequentially determined using Napari(9) and CATM, respectively.

Context-aware template matching (CATM)
To enhance molecular assignment within crowded condensates, we developed a context-aware template matching (CATM) procedure. Following deep learning-based particle identification, CATM performs local template matching and clash resolution to accurately determine molecular positions and orientations. For clarity, in the description below we use the following terms: “particle” for a generic object placed at a position in the map, “model” for the low resolution filtered structure or experimentally determined structure, “template” for a model rotated to a particular orientation (used in clash resolution), and “reference” for the image of the template distorted according to missing wedge and CTF effects (used in CCC calculation).

<u>Template generation</u>
In the CATM pipeline, tomograms reconstructed in Warp through back projection were low-pass filtered with Gaussian filter at 25 Å using EMAN2 (10), and a corresponding CTF model for each tomogram was generated. An initial nucleosome model was generated by low-pass filtering a nucleosome structure (pdb 6pwe) to 25 Å resolution with 8 Å/pixel to match the experimental data. The model was subsequently replaced with an experimental structure determined by subtomogram averaging to achieve better fitting for all the results reported here. Missing wedge and CTF artifacts severely distort the nucleosome structure in tomograms (Fig. 3a, SI Appendix, Fig. S3). It was essential to properly account for these distortions during template matching, so that the references better represented the real data. We took two steps in this regard. First, the nucleosome model was applied to the 3D CTF model generated from Warp to assess the distortion and choose the contour level of the distorted template to minimize the artifacts. In addition, for a given nucleosome model we created a library of templates representing different orientations of the nucleosome, uniformly sampled in 3D space. Each template was then appropriately corrected for the missing wedge effect and CTF modulation to produce a library of references for use in CCC calculations during template matching as described below.

<u>Local template matching</u>
Coordinates derived from the segmentation phase were used to extract subtomograms. For each subtomogram, the program iterates through the library of reference images representing different

orientations of the model, calculating CCC for each to the raw tomogram via fast Fourier transformation, similar to methodology implemented in PyTom. In this procedure, particle centers of mass are scanned through all voxels within a 4-voxel radius of the centroids identified in the segmentation phase. At each voxel, CCC was calculated for the library of references, based on an internal mask created by binarizing the template and extending it for three voxels in each dimension. References with CCCs greater than a cutoff (typically 0.3) were saved for each candidate voxel to facilitate clash resolution (see below). Each subtomogram is considered a particle in the class resolution procedures below, and references (and their corresponding templates) were ranked by their CCCs in descending order.

Clash resolution

Following template matching, the list of particles was ranked by their maximum CCCs, and then mapped back to the tomogram sequentially starting with the template whose reference afforded the highest CCC for the particle. In cases where a clash occurred during new particle placement, the other templates for the new particle (corresponding to references with CCC values less than the maximum) were then used to resolve the clash. If the clash could be resolved by another template, the two templates were then saved in an assigned list and mapped to an assigned map. Otherwise, a mechanism was employed to resolve the clash that began by retrieving the nearest neighbors of the pair among the currently mapped particles. The densities of the nearest neighbors were then erased from the assigned map, and a subregion of the assigned map (80 voxels) was subtracted from the assigned map to accelerate template optimization. Within this dense environment, all possible combinations of templates corresponding to both particles were sampled to find a clash-free solution whose references afforded the highest combined CCC value. If a clash could not be resolved—typically due to false positives in segmentation—the particle with the highest CCC value was retained. The process of particle placement and clash resolution continued until all particles identified by segmentation had been accounted for. Note that in addition to placing particles in order of CCC values, we also examined random ordering or spatially defined ordering. Both of these alternatives yielded less random nucleosome orientation distributions than the CCC-based procedure, indicating poorer performance.

The process concluded with the generation of various electron microscopy (EM) format coordinate files, alongside an assigned particles tomogram file, documenting the final particle placements and orientations.

Benchmarking of template matching algorithms

To evaluate the efficacy of various template matching algorithms, we employed cryotomosim (11) to simulate tomograms, using a nucleosome structure (PDB 6pwe) low-pass filtered to 25 Å. This structure was randomly rotated and embedded in a 3D volume alongside DNA fragments of 25 base pairs, equivalent in number to the nucleosomes, to mimic the environment of experimental tomograms. Vitrified ice simulated effects were added, and tilt series were created under specific conditions(11): 300 keV voltage, spherical aberration of 2.7, sigma of 0.9, defocus of -4 μm, total dose of 150 electrons/Å^2, symmetric tilt pattern, pixel size of 8 Å, and a tilt range from -60° to 48° with 2° increments. Subsequent tomograms were reconstructed for analysis.

A soft mask with two voxels of extension beyond the density of the nucleosome template, plus two voxels of soft edge decay was created in Relion and systematically used across all tested software.

Standard Template Matching (TM) algorithm

Reference images corresponding to templates at various angles, and distorted with missing wedge effects, were matched against the tomogram using the same cross-correlation function as employed in CATM. The highest CCC value and its corresponding orientation for each voxel in the tomogram were recorded. Particles were ranked based on CCCs and filtered to remove those within 5 voxels (4 nm) of each other. Remaining templates were mapped back to the tomogram, removing any that clashed (defined by two templates occupying the same voxel in the tomogram). Various CCC cut-offs were used to define particle sets, which were then used to calculate metrics

for the benchmark, including precision, recall and F1 score (defined below) by comparison to the ground truth, and angular distribution.

Context-Aware Template Matching (CATM)
For CATM testing, neural network weights obtained from real data segmentation with DeepFinder were used to segment the simulated data without additional training. The segmented map provided centroids for particle identification, which were then used in CATM as described above.

Template matching in Pytom
Version 0.971 of Pytom was applied to the same template and mask as TM and CATM. The sampling angular interval was set to 12.85°, and wedge correction was applied to the template. Other parameters were used at their default values. Templates were filtered by a 5-pixel distance cut-off in Pytom, and only non-clashing templates were saved for analysis.

Performance metric calculation
Performance was measured for each approach using mechanisms similar to the Shrec 2019 cryo-ET Classification benchmark (12). For positional accuracy, a particle was considered a True Positive (TP) if its predicted centroid was within 4 nm of the ground truth. For orientation accuracy, a vector perpendicular to the nucleosome plane was defined in the reference frame, and orientation was considered a TP if the angle between the predicted and ground truth vectors was < 30°. True Positives (TP) were defined as particles correctly assigned; False Positives (FP) as particles assigned by the program but not present in the ground truth; and False Negatives (FN) as ground truth particles not assigned by the program. Precision was calculated as TP/(TP+FP), recall as TP/(TP+FN) and the F1 score as the harmonic mean of precision and recall.

$$F1 = 2 * \frac{Precision * Recall}{Precision + Recall}$$

Subtomogram averaging using Warp-Relion-M
Following CATM, the coordinates and orientation data of the particles were used to reconstruct subtomograms at an 8 Å/pixel resolution in Warp. These subtomograms were subsequently averaged and assembled into an initial model, which was then low pass filtered to 40 Å and used as a reference for further refinement in Relion. Particle refinement involved enhancing precision iteratively while down sampling to 6 Å/pixel, 4 Å/pixel, and finally 2 Å/pixel. This progressive refinement ensured increased resolution and accuracy of the particle models. After completing the final refinement step, the particles were imported into M software(13) for further pose and contrast transfer function (CTF) refinement. This multi-step process leverages the strengths of Warp for initial reconstruction and Relion for detailed refinement, culminating in M for final adjustments, thus optimizing the accuracy and resolution of the resulting structural models.

For the reconstituted chromatin samples and isolated HeLa cell nuclei, averaging was performed for all assigned nucleosomes by CATM; none were discarded. For the NIH3T3 cells sample, the particles were then separated into two different classes, one giving 12 Å resolution for 6470 particles, and a second giving 22 Å resolution for 6804. Resolution estimation was performed with Fourier shell correlation in Relion.

Analysis of Vitrobot and Chameleon AWI
The air-water interface (AWI) was manually defined for each tomogram with control points using IMOD (14). These were used to create a 2D triangulation mesh using MATLAB and Dynamo scripts (15). For each nucleosome particle, a distance and relative orientation to the AWI was calculated. The relative orientation was taken as the absolute value of the dot product between the surface normal of the closest AWI mesh face and a vector normal to the nucleosome face. To assess preferred orientation at the AWI, the orientation of nucleosomes within 20 nm of the AWI were assessed relative to those further than 20 nm. Oriented nucleosomes were visualized at their native positions within the tomogram using ChimeraX (16) and the ArtiaX plug-in (17).

Analysis of the nucleosome orientation distribution

In our coordinate system the origin is set to the centroid of the reference nucleosome, which was approximated as having 2-fold symmetry about the dyad axis (i.e. composed of palindromic DNA); the Z-axis is perpendicular to the nucleosome plane, the X-axis extends from the centroid to the nucleosome dyad, and the Y-axis is orthogonal to the X-Z plane. For each nucleosome assigned by template matching we determined the angle between its Z-axis and the beam direction or the two beam-normal directions. Since the angle between a random vector and a plane follows a sinusoidal distribution (18) a random arrangement of nucleosomes will exhibit a similar sinusoidal distribution of these angles.

Analysis of nucleosome orientation and clustering at the condensate-buffer Interface
To analyze nucleosome organization at the condensate-buffer interface, we first delineated the interface every 10 slices in the tomogram using IMOD (14). We then applied IMOD's linear interpolation to extend the annotation across the entire tomogram. The segmented surfaces were then used to reconstruct the entire droplet sphere with least squares regression (we note that two-dimensional projections of condensates observed in the lamella at low magnification were circular (Fig. 2d), justifying the assumption of spherical shape of the entire structure). For each assigned nucleosome, we calculated the shortest distance to the interface and identified the corresponding surface anchor points. Surface normals were determined using the normalized vector from the sphere center to each surface point. Nucleosomes within 20 nm of the interface were classified as the "Surface" group. To assess nucleosome orientation, we measured the angle between each nucleosome's plane normal and the nearest condensate-buffer interface normal, both for the "Surface" group and all nucleosomes.

For nucleosome clustering analysis within the condensate, we employed DBSCAN, a density-based clustering algorithm. The algorithm iterates through nucleosomes in the tomogram, identifying clusters by examining their local neighborhoods. Densely packed nucleosomes were grouped into clusters, while sparsely distributed ones were treated as noise. The maximum neighborhood radius was set to 12 nm, and nucleosomes with at least four neighbors within this radius were considered core points.

Reconstruction of the condensate graph network and calculation of the entropy of valence distribution
To construct the nucleosome networks, particle coordinates were imported using an in-house script, and the geometric graph network was reconstructed using networkX (19). Each particle was treated as a node, and edges were assigned between nodes when the distance between nucleosome centers of mass was less than 12 nm. The entropy of valence (H in bits) distribution measures the diversity of the degree distribution (20). It is defined as:

$$H = -\sum p(k) \log_2 p(k)$$

Where p(k) is the probability of a node having degree k. The maximum possible entropy (Hmax) depends on the number of degree values K in the network (K is 6 for both reconstituted and native chromatin) and for a network with K possible degrees:

$$H_{max} = \log_2 K$$

For comparison between samples, the normalized entropy ($H_{normalized}$) is calculated by:

$$H_{normalized} = \frac{H}{H_{max}}$$

Four tomograms were used to calculate the average and standard deviation of the $H_{normalized}$.

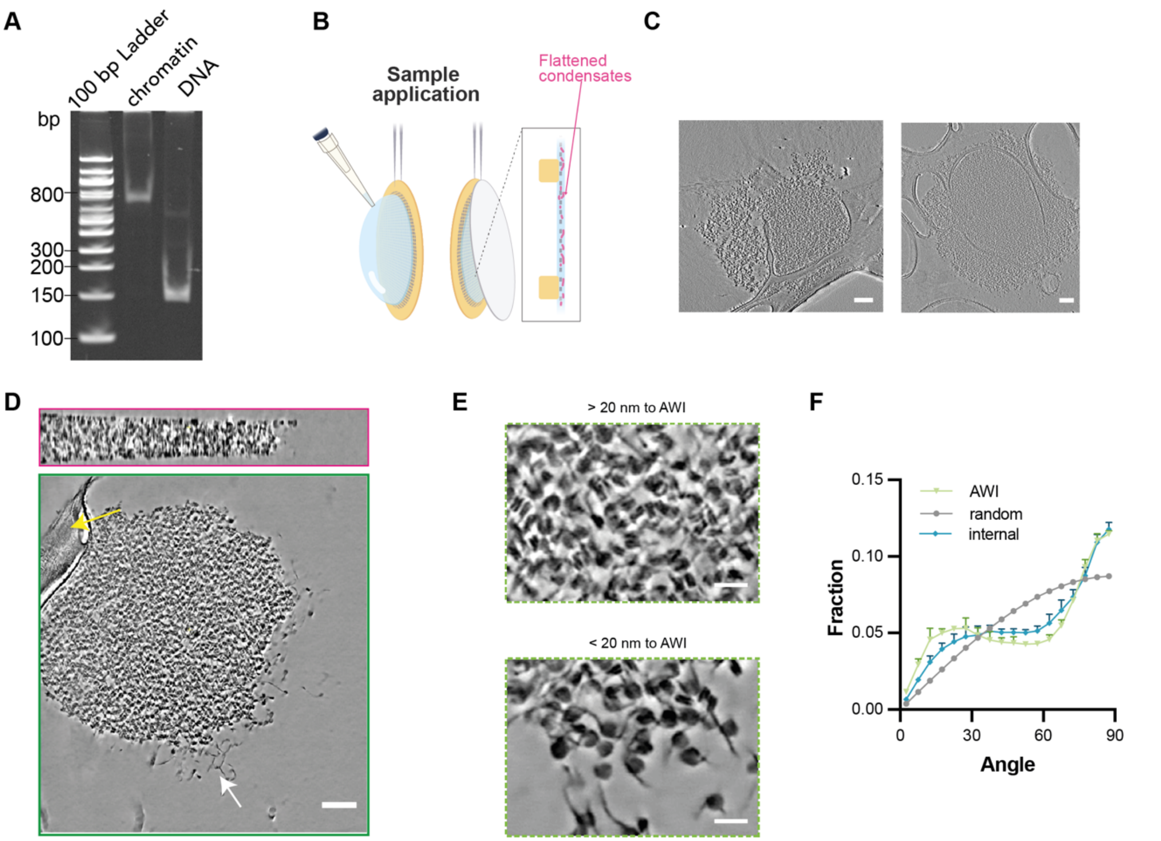

Fig. S1. Disruption of condensate structures by back blotting and self-wicking methods.
(A) A Native PAGE electrophoretic mobility shift assay, stained with Ethidium Bromide, was performed to analyze the 12x601 array DNA both in its free form and after chromatin assembly. The arrays were digested with *Bst* XI, which cleaves them into single 601 arrays before the assay.
(B) Diagram depicting the process of sample application followed by back blotting.
(C) Representative tomographic slices of chromatin condensates prepared using the back blotting method. Scale bar is 100 nm.
(D) Orthogonal cross-sections of chromatin condensates processed using the self-wicking method. Yellow arrow indicates carbon support film on the grids. White arrow indicates exposed nucleosomes and bare DNA regions. Scale bars are 100 nm. X-Y, X-Z views are shown with green, magenta outlines, respectively.
(E) Representative sections demonstrating chromatin condensates (prepare with self-wicking method) located close to and away from the air-water interface. Scale bars are 20 nm.
(F) Angular distribution between the normal to the nucleosome plane and the plane of AWI at the AWI and in the core of the condensate prepared with self-wicking method.

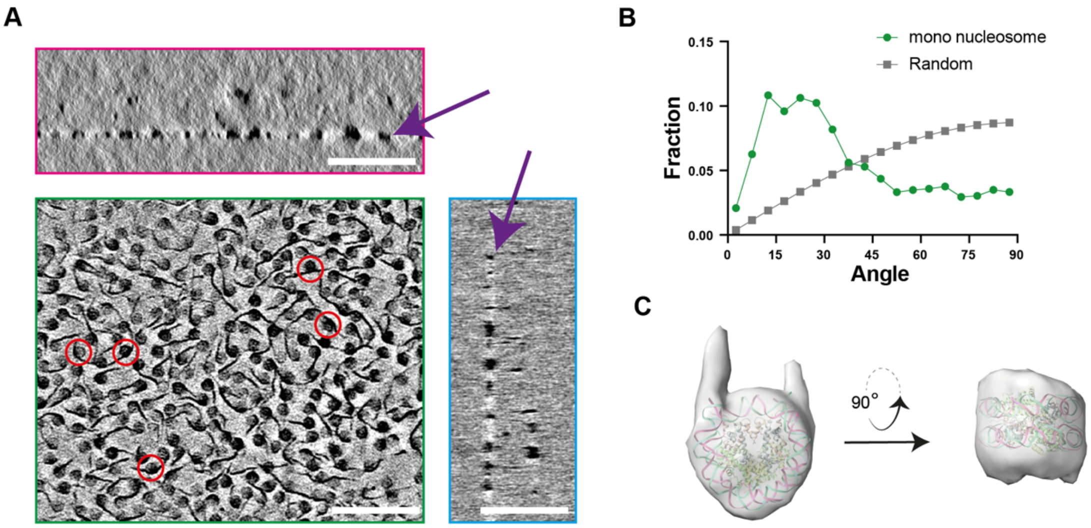

Fig. S2. Denaturation and orientational bias of mononucleosomes at the air-water interface with blotting.

(A) Orthogonal cross-sections from a reconstructed tomogram with mononucleosomes prepared with blotting. Purple arrows indicate the air-water interface. X-Y, X-Z and Y-Z views are shown with green, red and blue outlines, respectively. Red circles indicate some unwrapped nucleosomes. Scale bar is 100 nm.

(B) Distribution of angle between the normal to the nucleosome plane and the beam direction (Z-axis) as determined by CATM of the 4705 mononucleosomes observed in the tomograms.

(C) Subtomogram average structure of the mononucleosome from 4705 particles.

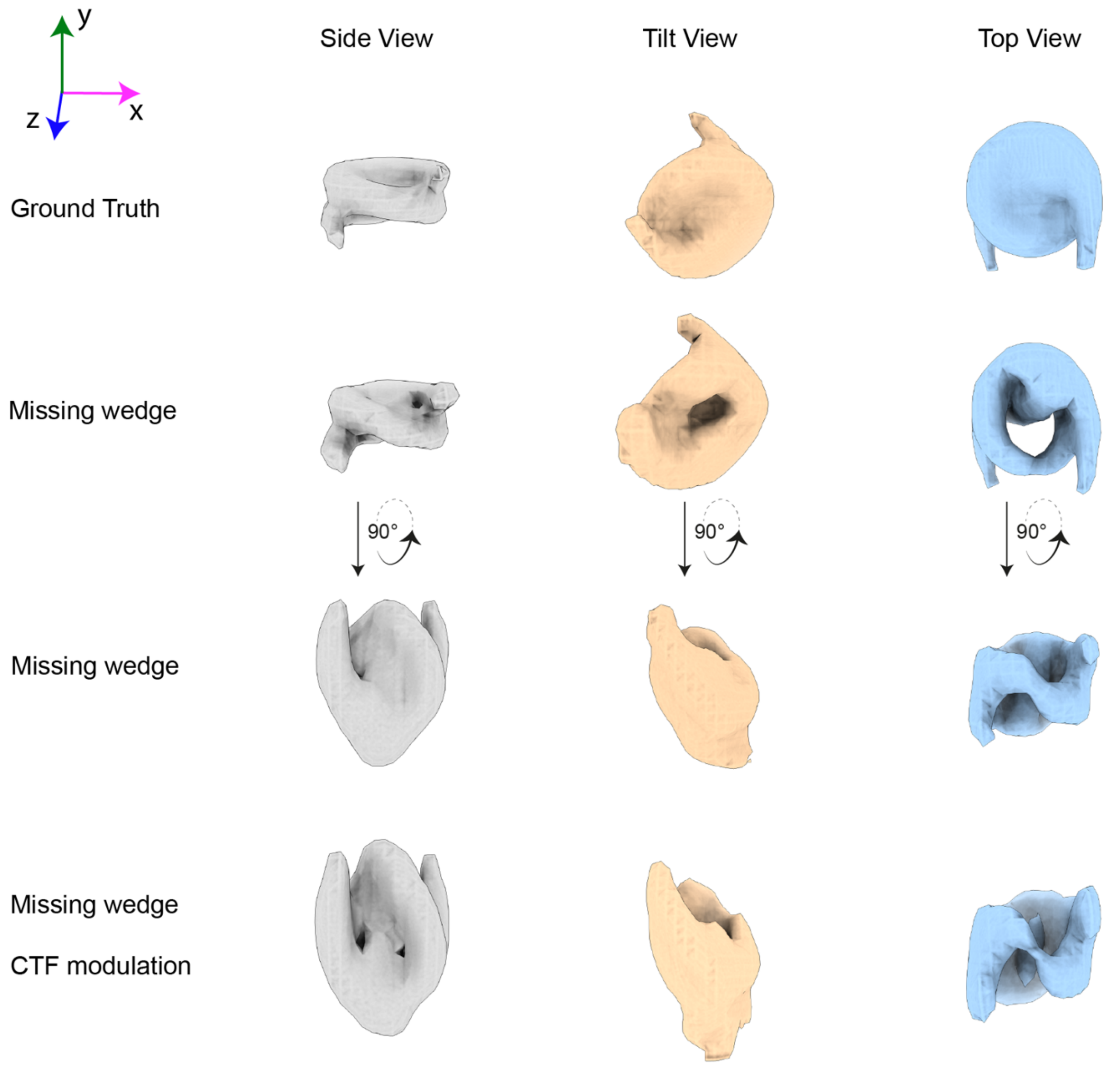

Fig. S3. Missing wedge and CTF modulation distort the nucleosome.
The top panel displays a nucleosome structure from various perspectives. The missing wedge effect was then applied to nucleosomes in different orientations, causing pronounced distortions along the z-axis, as depicted in the bottom two panels. Additionally, modulation by the contrast transfer function (CTF) further exacerbates the distortion, introducing more artifacts into the nucleosome images.

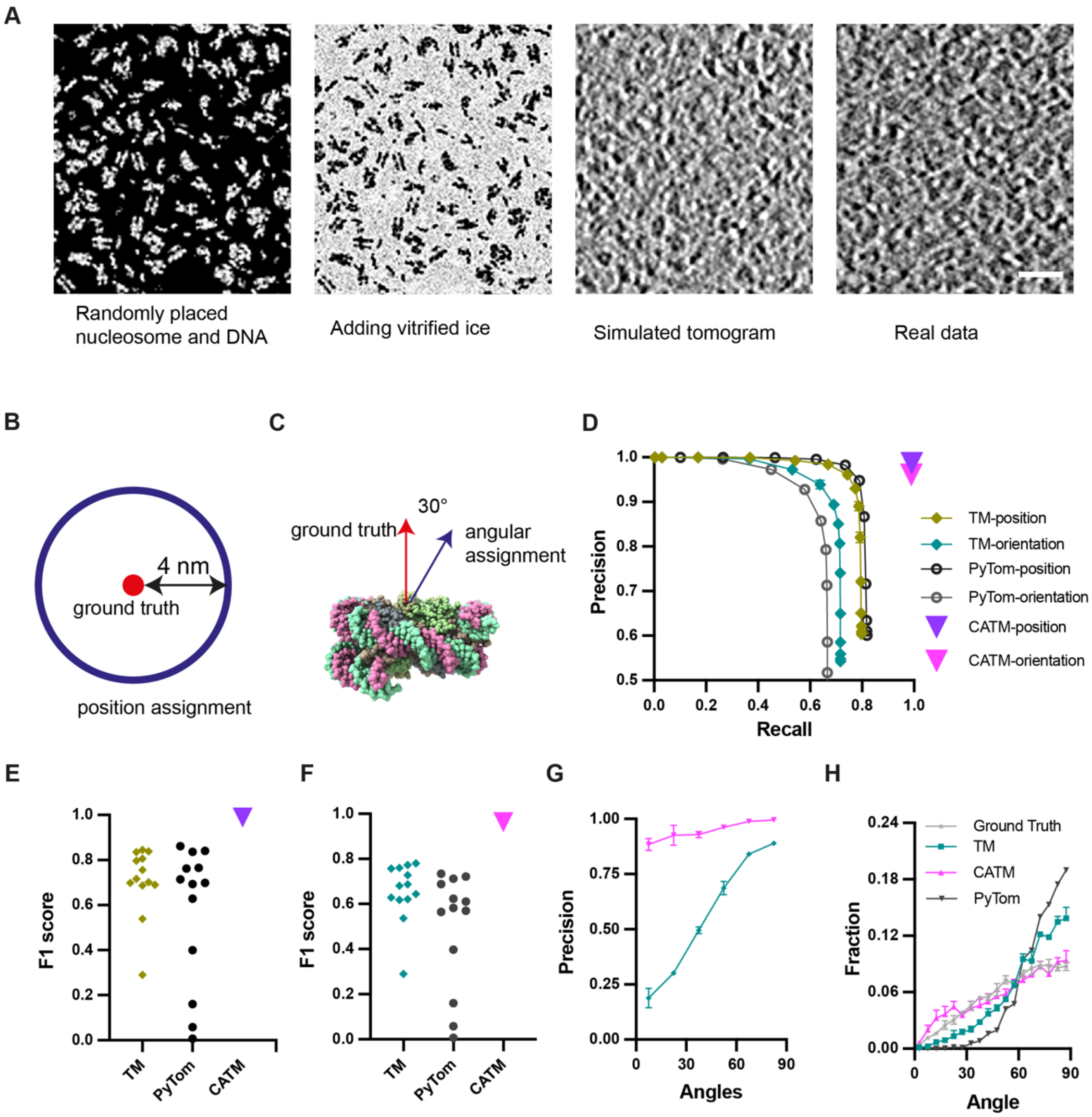

Fig. S4. Benchmark of CATM against standard template matching (TM) and PyTom template matching.

(A) Simulation of tomograms. Nucleosomes and 25 bp DNA were randomly placed in a 3D volume at a density equivalent to chromatin condensates, and vitrified ice was simulated. The volume was then subjected to tilt-series simulation and tomogram reconstruction. The tomograms was low pass filtered to 25 Å and compared with real chromatin data with the same filter. Scale bar is 20 nm.

(B) Particles with center of mass assigned within 4 nm of the center of mass of a ground truth nucleosome are considered accurate.

(C) For orientation accuracy, a vector perpendicular to the nucleosome plane was defined. The angle between the vectors from the ground truth and assigned nucleosomes was calculated. Predictions within 30° were considered accurate.

(D) Comparative performance in nucleosome assignment by CATM, TM (see Standard Template Matching Algorithm methods) and PyTom. For TM and PyTom, with various of cross-correlation cutoffs the algorithms have different performance. CATM produces only a single set of assignments, error bars for CATM are smaller than the symbols.

(E-F) F1 scores of position (e) and position+orientation (F) for the different algorithms.

(G) Orientation assignment precision as a function of orientation with respect to the beam direction (Z-axis) for each method. Zero degrees is the top view of a nucleosome (disc oriented perpendicular to beam direction), 90 degrees is a side view.
(H) Distribution of nucleosome orientations with respect to the beam direction (Z-axis) as determined by CATM, TM and PyTom. Nucleosomes are oriented randomly in the ground truth (gray line), producing a sinusoidal distribution (18).

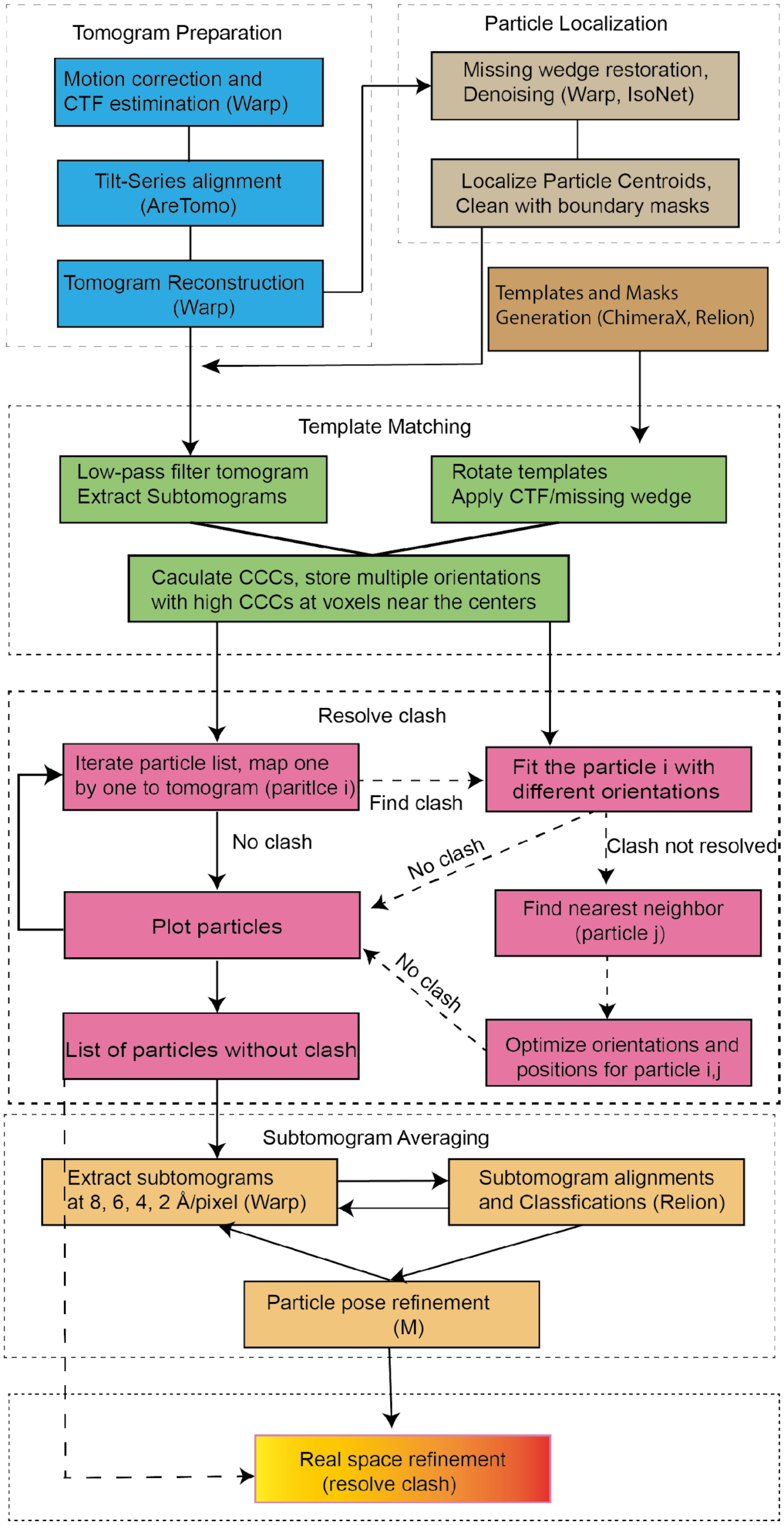

Fig. S5. CATM data analysis pipeline.

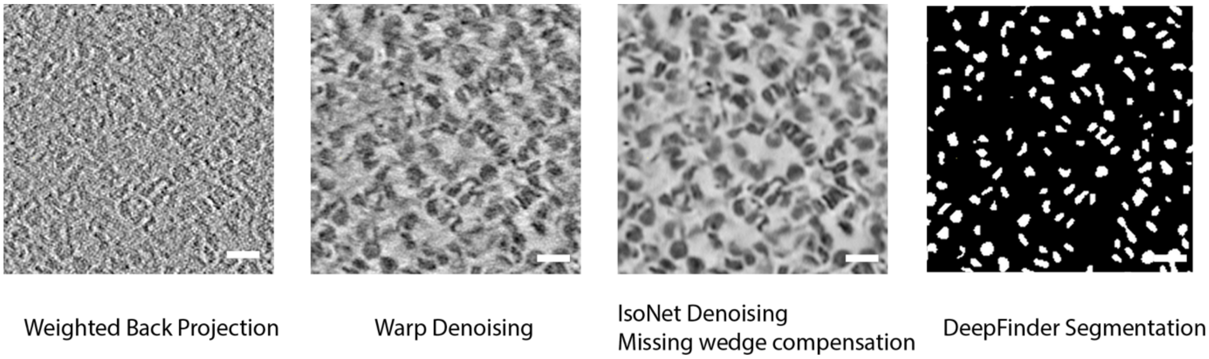

Fig. S6. Tomogram denoising, missing wedge compensation, and segmentation.
Representative slices of tomogram generated by weighted back projection, Warp and IsoNet denoising , missing wedge compensation, and the DeepFinder segmentation. Scale bar is 20 nm.

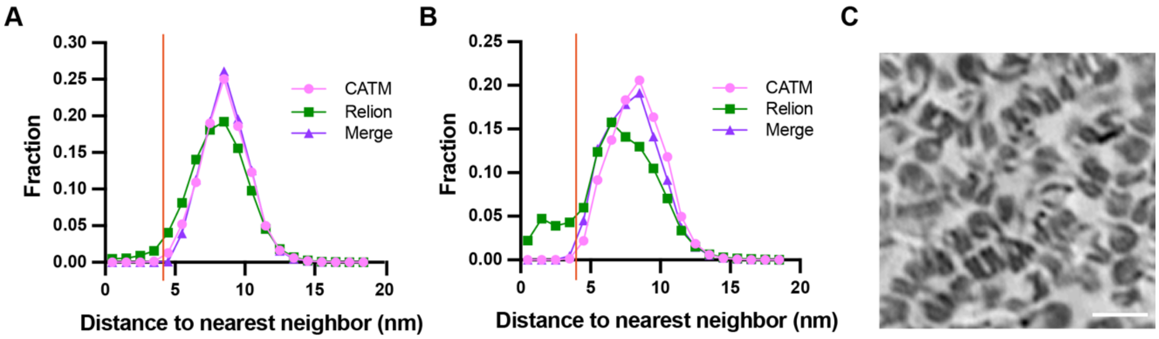

Fig. S7. Relion tends to refine particles into local minima

(A-B)Quantification of the nearest neighbor distance for CATM, Relion and the subsequent refined particles which merge the CATM and Relion refinement for chromatin condensate (A) and another case of chromatin condensates enriched in stacking nucleosomes (B). The yellow vertical line indicates the nucleosome distance tolerance of 4 nm, slightly smaller than the width of the nucleosome disk.

(C) A representative image of the condensate with highly abundant stacking.

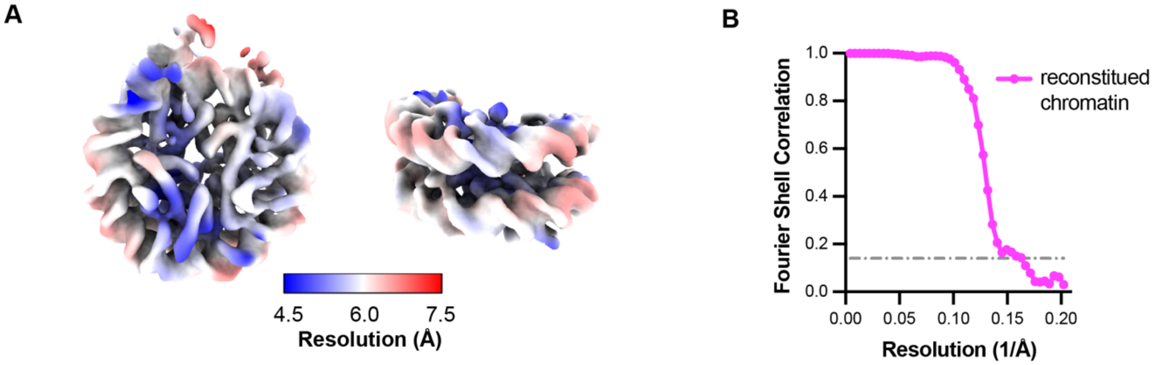

Fig. S8. Resolution estimation for subtomogram averaging with FSC.
(A) Subtomogram averaging to reconstruct a nucleosome structure from reconstituted chromatin condensates, colored by local resolution.
(B) Fourier shell correlation between two independently processed halves of the data set used in reconstituted chromatin condensates.

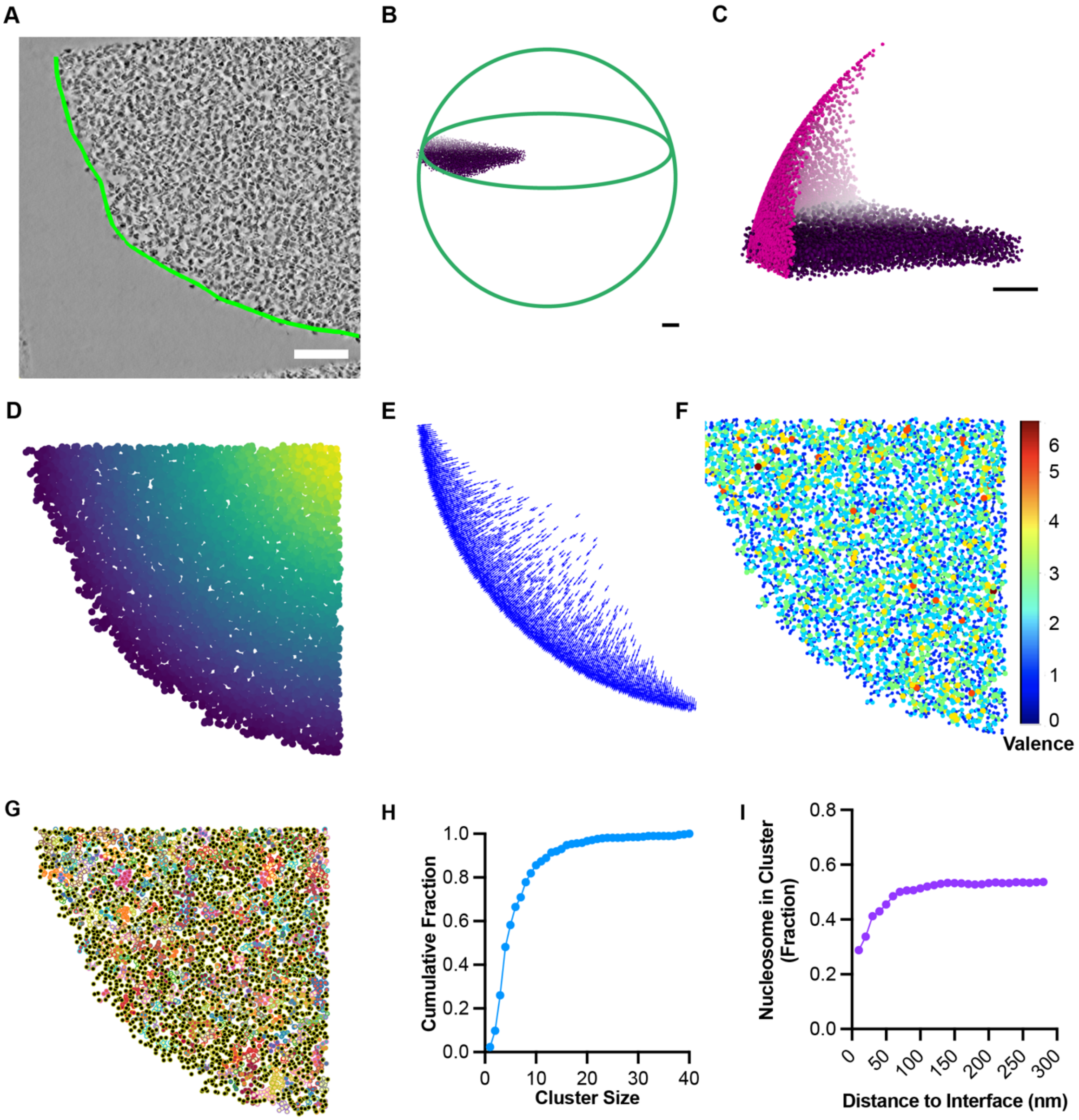

Fig. S9. Additional example of spatial organization of nucleosomes within a chromatin condensate.

(A) Manually traced condensate-buffer interface (green lines) on a slice of the represented tomogram, scale bar is 100 nm.

(B) Orthogonal view of a lamella with assigned nucleosomes (purple dots), and the full condensate sphere fit to its surface (green lines).

(C) Dots plot of nucleosomes in the lamella (purple) with the points on the condensate surface that are closest to at least one nucleosome indicated in magenta.

(D) Heatmap of nucleosome positions (dots), colored by their shortest distance to the interface.

(E) Surface normals of the condensate, represented by blue arrows.

(F) Chromatin condensate network graph, where each node represents a nucleosome (not to scale) from panel (a), color-coded by its interaction valence.

(G) Nucleosome clusters within the condensate, with distinct colors indicating different clusters. Black dots represent nucleosomes not assigned to any cluster.

(H) Cumulative distribution of cluster sizes (number of nucleosomes) from panel (F).

(I) Fraction of nucleosomes within clusters as a function of their distance from the condensate interface, based on panels (D) and (G).

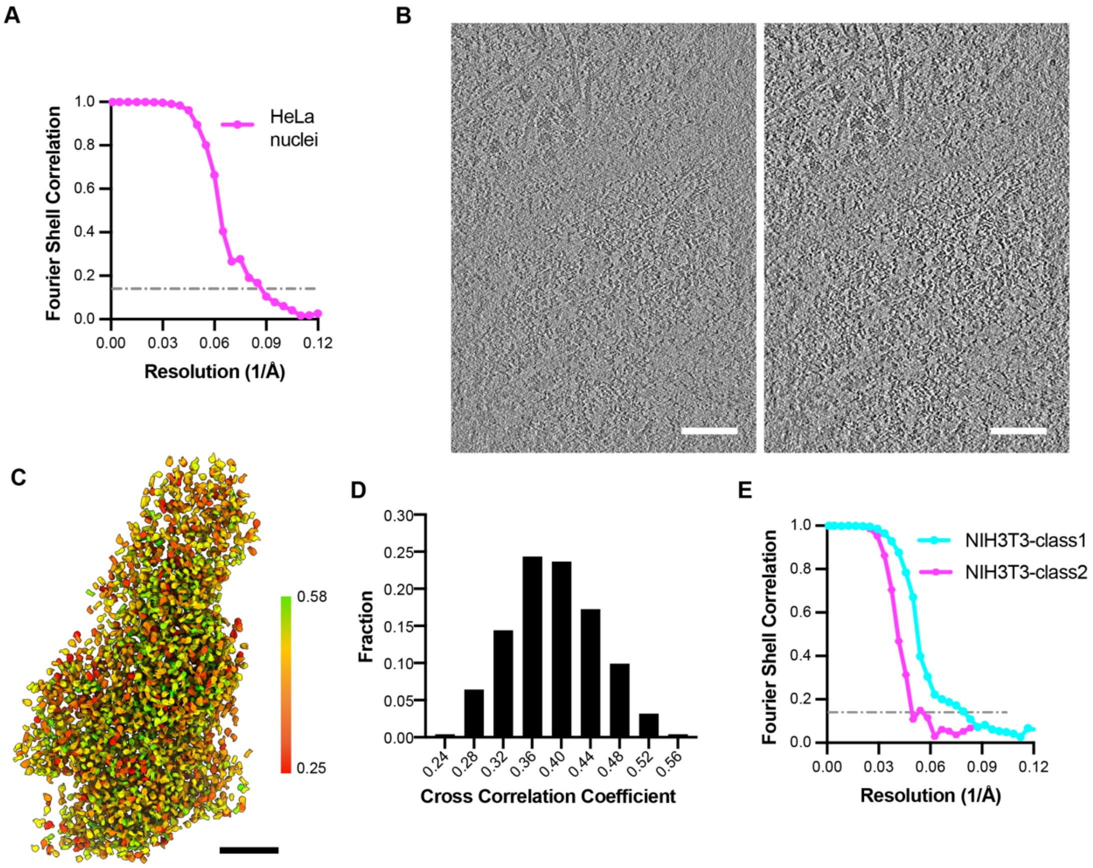

Fig. S10. Analysis of tomograms from native chromatin
(A) Fourier shell correlation between two independently processed halves of the data set used in HeLa nuclei.
(B)Cross-sections from a tomographic volume of an NIH3T3 cell. The left panel shows the raw back-projection, while the right panel displays the low-pass filtered version at 25 Å.
(C) Nucleosome models assigned within the tomogram (B), color-coded based on their corresponding cross-correlation coefficient as determined by CATM.
(D) Frequency distribution of the cross-correlation coefficients from the assignment in (B).
(E) Fourier shell correlation between two independently processed halves of the data set used in NIH3T3 tomograms.

Table S1: **cryo-ET data collection and reconstruction statistics**

	Vitrobot-prepared chromatin	Chameleon prepared	HPF prepared Reconstituted chromatin	Nuclei chromatin	Cellular chromatin
**Data collection**					
Magnification	33 K	33 K	81 K	81 K	64 K
Voltage (kV)	300	300	300	300	300
Defocus range (μm)	-0.5	-0.5	-3 to -4.5	-3 to -4.5	-4
Pixel size (Å)	2.06	2.06	1.516	1.516	1.34
Electron exposure (e–/Å^2)	150	150	178	178	140
Tilt-range/step (°)	-60,+60 / 2	-60,+60 / 2	-48,+60 / 2	-48,+60 / 2	-54,+54 / 3
Tilt-scheme	dose-symmetric, grouping 2		dose-symmetric, grouping 3		dose-symmetric, grouping 1
**Processing**					
Symmetry imposed	-	-	C1	C1	C1
Initial particle images (no.)	-	-	126125	35503	13277
Final particle images (no.)	-	-	126125	35503	Class 1: 6470<br>Class 2: 6804
Map resolution (Å)	-	-	6.1	12	Class1: 12<br>Class2: 22
FSC threshold	0.143				

Movie S1 (separate file). To preserve chromatin condensates in their native state, we used high-pressure freezing and cryo-FIB milling (the Waffle method (21)) to prepare lamellae. The chromatin droplet solution was applied to the back side of the cryo-EM grid and rapidly vitrified using a high-pressure freezer. The grid was then imaged with a cryo-fluorescence microscope to localize the chromatin condensates. Subsequently, the grid was transferred to a focused ion beam scanning electron microscope (cryo-FIB-SEM) to remove excess material and produce thin lamellae. After bulk milling, notch milling (3, 21) was performed to ensure lamellae stability. Thin lamellae (<150 nm) were critical for achieving high image quality. The prepared lamellae were then transferred to a cryo-electron microscope, where tilt-series were collected using a dose-symmetric scheme. Finally, the tomograms were reconstructed via back-projection, and nucleosomes were properly assigned using our algorithm.

Movie S2 (separate file). In the crowded condensate environment, molecules are densely packed, making it challenging to determine the exact number of nucleosomes, as seen with two stacked nucleosomes in this example. To address this, we tilt the sample and collect tilt-series at various angles. However, due to stage limitations, some angles cannot be sampled, resulting in a missing wedge of information. Combined with contrast transfer function (CTF) modulation, this distortion elongates particles along the beam direction, complicating their identification. Here, we compare standard template matching (TM) with our context-aware template matching (CATM). In TM, the program searches for the best fit at local minima, excluding particles based on distance metrics, which often leads to errors in particle number, orientation, or both. In contrast, CATM uses a more robust pipeline: first, particle centroids are identified using deep learning-based segmentation or picking. Local template matching is then performed around these centroids, recording multiple orientation and position possibilities for each particle. Finally, particles are mapped back into the tomogram, and potential clashes are resolved by optimizing their orientation combinations. This approach significantly improves accuracy in identifying both the number and orientation of particles.

SI References

1. B. A. Gibson *et al.*, Organization of Chromatin by Intrinsic and Regulated Phase Separation. *Cell* **179**, 470-484.e421 (2019).
2. K. Kelley *et al.*, Waffle Method: A general and flexible approach for improving throughput in FIB-milling. *Nature Communications* **13**, 1857 (2022).
3. O. Klykov *et al.*, In situ cryo-FIB/SEM Specimen Preparation Using the Waffle Method. *Bio Protoc* **12** (2022).
4. M. Schorb, I. Haberbosch, W. J. H. Hagen, Y. Schwab, D. N. Mastronarde, Software tools for automated transmission electron microscopy. *Nat Methods* **16**, 471-477 (2019).
5. F. Eisenstein *et al.*, Parallel cryo electron tomography on in situ lamellae. *Nature Methods* **20**, 131-138 (2022).
6. D. Tegunov, P. Cramer, Real-time cryo-electron microscopy data preprocessing with Warp. *Nature Methods* **16**, 1146-1152 (2019).
7. S. Zheng *et al.*, AreTomo: An integrated software package for automated marker-free, motion-corrected cryo-electron tomographic alignment and reconstruction. *Journal of Structural Biology: X* **6**, 100068 (2022).
8. B. A. Lucas, N. Grigorieff, Quantification of gallium cryo-FIB milling damage in biological lamellae. *Proc Natl Acad Sci U S A* **120**, e2301852120 (2023).
9. N. Sofroniew *et al.* (2024) napari: a multi-dimensional image viewer for Python. (Zenodo).
10. M. Chen *et al.*, A complete data processing workflow for cryo-ET and subtomogram averaging. *Nat Methods* **16**, 1161-1168 (2019).
11. C. Purnell *et al.*, Rapid Synthesis of Cryo-ET Data for Training Deep Learning Models. *bioRxiv* (2023).
12. I. Gubins *et al.* (2019) Classification in Cryo-Electron Tomograms. eds S. Biasotti, G. Lavoué, R. Veltkamp (The Eurographics Association).
13. D. Tegunov, L. Xue, C. Dienemann, P. Cramer, J. Mahamid, Multi-particle cryo-EM refinement with M visualizes ribosome-antibiotic complex at 3.5 Å in cells. *Nature Methods* **18**, 186-193 (2021).
14. D. N. Mastronarde, S. R. Held, Automated tilt series alignment and tomographic reconstruction in IMOD. *Journal of Structural Biology* **197**, 102-113 (2017).
15. D. Castano-Diez, M. Kudryashev, M. Arheit, H. Stahlberg, Dynamo: a flexible, user-friendly development tool for subtomogram averaging of cryo-EM data in high-performance computing environments. *J Struct Biol* **178**, 139-151 (2012).
16. E. C. Meng *et al.*, UCSF ChimeraX : Tools for structure building and analysis. *Protein Science* **32**, e4792 (2023).
17. U. H. Ermel, S. M. Arghittu, A. S. Frangakis, ArtiaX : An electron tomography toolbox for the interactive handling of s ub-tomograms in UCSF ChimeraX. *Protein Science* **31** (2022).
18. J. Singh, J. M. Thornton, The interaction between phenylalanine rings in proteins. *FEBS Letters* **191**, 1-6 (1985).
19. A. A. Hagberg, D. A. Schult, P. J. Swart (2008) Exploring Network Structure, Dynamics, and Function using NetworkX. in *Python in Science Conference*, pp 11-15.
20. R. V. Solé, S. Valverde, "Information Theory of Complex Networks: On Evolution and Architectural Constraints" in Complex Networks*,* E. Ben-Naim, H. Frauenfelder, Z. Toroczkai, Eds. (Springer Berlin Heidelberg, Berlin, Heidelberg, 2004), pp. 189-207.
21. K. Kelley *et al.*, Waffle Method: A general and flexible approach for improving throughput in FIB-milling. *Nat Commun* **13**, 1857 (2022).
